# Mussel adhesion is dictated by time-regulated secretion and molecular conformation of mussel adhesive proteins

**DOI:** 10.1038/ncomms9737

**Published:** 2015-10-28

**Authors:** Luigi Petrone, Akshita Kumar, Clarinda N. Sutanto, Navinkumar J. Patil, Srinivasaraghavan Kannan, Alagappan Palaniappan, Shahrouz Amini, Bruno Zappone, Chandra Verma, Ali Miserez

**Affiliations:** 1 School of Materials Science and Engineering, Nanyang Technological University, 50 Nanyang Avenue, Singapore 639798, Singapore; 2 Centre for Biomimetic Sensor Science, Nanyang Technological University, 50 Nanyang Drive, Research Techno Plaza, XFrontiers Block, Singapore 637553, Singapore; 3 School of Biological Sciences, Nanyang Technological University, 60 Nanyang Drive, Singapore 637551, Singapore; 4 Dipartimento di Fisica, Università della Calabria, 87036 Arcavacata di Rende (CS), Italy; 5 Bioinformatics Institute A*Star, 30 Biopolis Street, Singapore 138671, Singapore; 6 Consiglio Nazionale delle Ricerche, CNR-Nanotec, UOS Licryl-Cosenza, 87036 Rende (CS), Italy; 7 Department of Biological Sciences, National University of Singapore, 14 Science Drive 4, Singapore 117543, Singapore

## Abstract

Interfacial water constitutes a formidable barrier to strong surface bonding, hampering the development of water-resistant synthetic adhesives. Notwithstanding this obstacle, the Asian green mussel *Perna viridis* attaches firmly to underwater surfaces via a proteinaceous secretion (byssus). Extending beyond the currently known design principles of mussel adhesion, here we elucidate the precise time-regulated secretion of *P. viridis* mussel adhesive proteins. The vanguard 3,4-dihydroxy-L-phenylalanine (Dopa)-rich protein Pvfp-5 acts as an adhesive primer, overcoming repulsive hydration forces by displacing surface-bound water and generating strong surface adhesion. Using homology modelling and molecular dynamics simulations, we find that all mussel adhesive proteins are largely unordered, with Pvfp-5 adopting a disordered structure and elongated conformation whereby all Dopa residues reside on the protein surface. Time-regulated secretion and structural disorder of mussel adhesive proteins appear essential for optimizing extended nonspecific surface interactions and byssus' assembly. Our findings reveal molecular-scale principles to help the development of wet-resistant adhesives.

## Abstract (web summary)

Interfacial water constitutes a formidable barrier to strong surface bonding, hampering the development of water-resistant synthetic adhesives. Here, the authors elucidate the precise time-regulated secretion of mussel adhesive proteins in *Perna viridis*, probing their surface structures and subsequent roles.

## Graphical abstract

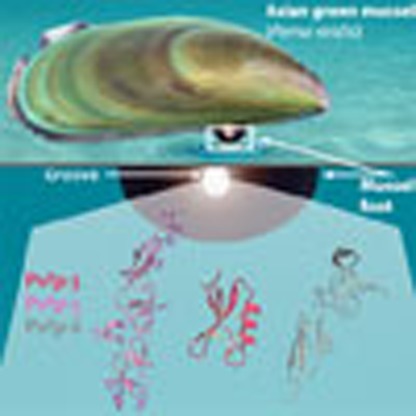

Water is regarded as a contaminant in adhesion technology because interfacial water leads to marked bond failure^1^,^2^,^3^. Despite this prevalent challenge, wave-swept rocky shores are home to a variety of sessile organisms that have evolved to attach themselves to submerged surfaces, forming dense communities, such as mussel beds, via self-organizing processes at the individual and the ecosystem level^4^,^5^,^6^,^7^. Mussels secrete a protein-based holdfast (byssus) strongly anchoring themselves to underwater solid substrates. The byssus distal end (byssal plaque) is specialized for adhesion, and six mussel foot proteins have been identified in the *Mytilus* genus (mfp-2, -3S, -3F, -4, -5 and -6). All mpfs are post-translationally modified to various extents with the amino acid 3,4-dihydroxyphenyl-L-alanine (Dopa)^1^,^8^,^9^. While initial studies have pointed out the definite role of Dopa in mussel adhesion^8^,^9^,^10^,^11^, driving intense research efforts to develop Dopa-containing polymers in applications such as wet adhesion promoters, medical sealants, self-healing polymers and anti-fouling coatings^12^,^13^,^14^,^15^, recent work has demonstrated that the success of mussel adhesion goes beyond the ‘Dopa paradigm'. These studies have notably revealed that redox interactions between mfps are key to maintaining Dopa adhesive activity^16^, that hydrophobic/hydrophilic interactions can also engage in adhesive interactions^17^,^18^ or that the local concentration of adhesive proteins during secretion also plays a critical role to ensure proper plaque delivery^19^. However, one important aspect that has eluded mussel adhesion research so far is the precise determination of adhesive proteins' secondary and tertiary structures, which are intrinsically related to their extensive nonspecific adsorption.

Combining RNA-seq with proteomic studies^20^, we have recently identified the byssal plaque proteins from the *Perna* genus. Three Dopa-containing foot proteins, termed Pvfp-3, -5 and -6, have been identified in the Asian green mussel *Perna viridis* (*P. viridis*), whose primary sequences share very low homology with mfps^20^,^21^. Both mfps and Pvfps comprise polymorphic families, with multiple genes and gene copies expressing different variants or processing diverse versions of mRNA via alternative splicing. Not all variants are eventually translated and biological variation is often observed among individuals^1^,^22^, with Pvfps spanning characteristic molecular weight in the range 4.5–6 kDa for Pvfp-3, 8–10.5 and 16–18 kDa for Pvfp-5, and a single protein of 11.4 kDa for Pvfp-6.

To fully understand mussel adhesion, it is essential to determine whether adhesive proteins are simultaneously discharged on surfaces as a complex matrix or deposited in distinct succession, as well as to establish the interplay between adhesive proteins' conformations in solution and protein–substrate interactions. Here we address these two aspects and establish that the secretion is precisely time regulated. Pvfp-5 is the first protein to initiate interaction with the substrate, displacing interfacial water molecules before binding to a model metal (hydr)oxide surface (TiO_2_ anatase) via coordination complex interactions. Using homology modelling and molecular dynamics simulations, we also highlight the role of adhesive proteins' three-dimensional (3D) structures in wet adhesion mechanisms. Notably, these modelling studies indicate that Pvfp-5 adopts a mostly elongated, unordered structure, whereby all Dopa residues are positioned on the protein surface to maximize surface interactions. However, Pvfp-5 is not fully disordered and structural stability is ensured through the presence of β-strand domains and disulphide bridges. Furthermore, Pvfp-5 contains multiple pairs of Dopa-lysine (Lys), which have recently been found to be critical in underwater adhesion^23^. These findings have the potential to further refine current developments in biomimetic adhesives. They can also inspire environmentally friendly fouling-resistant technologies aimed at preventing mussels from attaching to man-made underwater structures (for example, ship hulls and drilling platforms), decreasing frictional drag, fuel consumption and maintainance^24^.

## Results

### Time-resolved secretion of mussel adhesive proteins

**Figure 1:**
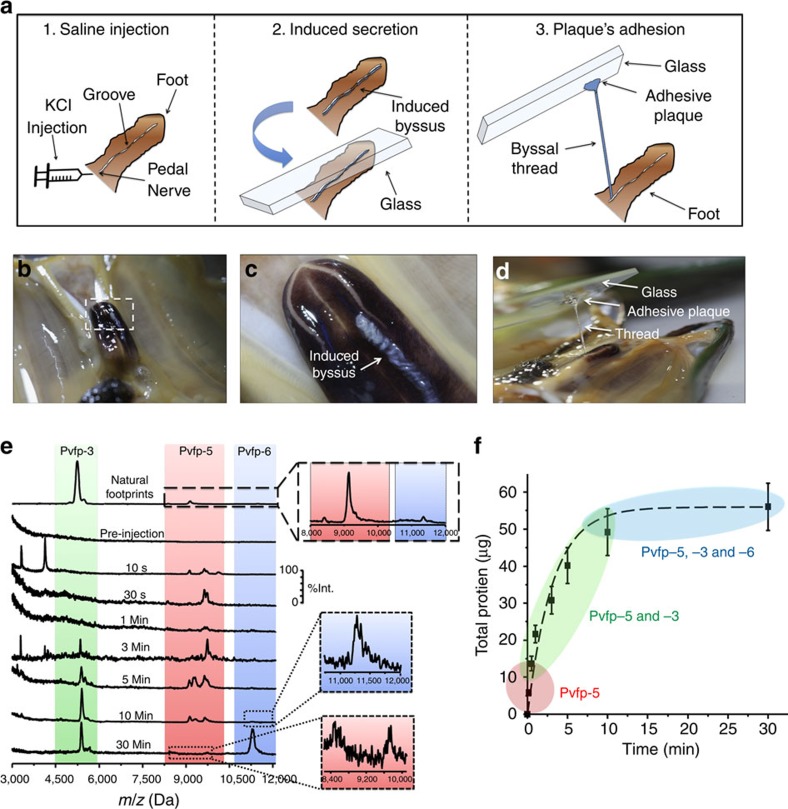
Mussel adhesive proteins are secreted at discrete time intervals. (**a**) Schematic of the injection of the saline solution to induce the secretion of functional mussel's byssus, as verified by the adhesion to glass via its plaque. (**b**) Mussel foot and (**c**) zoom in on the tip of the foot showing the freshly formed saline-induced byssal thread. (**d**) Adhesive plaque of the induced byssus adhering to a glass slide. The glued byssal thread was drawn out of the foot groove until the stem at the base of the foot. (**e**) MALDI-ToF MS spectra of native mussel footprint on glass (top spectrum), before saline injection (second from top) and at various time points (from 10 s to 30 min, bottom spectra) after injection. Coloured areas correspond to mass/charge (*m*/*z*) of Pvfp-3 (green), Pvfp-5 (red) and Pvfp-6 (blue). (**f**) Total proteins mass (±s.d.) secreted over time following KCl injection. Colour-coded areas denote the time intervals during which Pvfps are secreted (red: Pvfp-5; green: Pvfp-5 and -3; blue: Pvfp-5, -3 and -6). Dotted line is from a polynomial interpolation based on the average mass values. A total of 15 mussels were saline injected (see Supplementary Table 2 for full data).

The injection of KCl solution in the pedal nerve at the base of the mussel foot induced the secretion of a thread from the groove within the foot. When a glass slide was placed on top of the foot, the freshly secreted thread adhered to the glass via its adhesive plaque. The thread was also elastic and the plaque was firmly attached to the glass surface, demonstrating that the artificially induced secretion is a reasonable mimic of the natural byssus (Fig. 1a–d). Saline-induced adhesive secretions from mussels of *P. viridis* (*n*=15) were collected from the groove at the tip of the foot organ (Supplementary Fig. 1) before the saline injection and after injection at times ranging between 10 s and 30 min. The samples were analysed by matrix-assisted laser desorption/ionization time-of-flight mass spectrometry (MALDI-ToF MS) during the course of the secretion. Some differences were observed between specimens in terms of relative peak intensities and number of Pvfp variants. In most cases, a higher intensity was recorded for Pvfp-5 variants after 10 s, 5 min and 10 min (Fig. 1e), whereas in a few cases, Pvfp-5 variants were also observed after 30 min (Supplementary Fig. 2). While there was biological variation among specimens (Supplementary Table 1), a clear trend emerged with Pvfp-5 variants in the 8–10 kDa range always being secreted first, typically within 10 s after saline injection and before other Pvfps. Pvfp-3 variants in the 5–6 kDa range appeared 30 s after injection, often concurrently with Pvfp-5. Depending on the individual mussel, Pvfp-6 at 11.4 kDa appeared from 5 min after injection, along with Pvfp-3 and -5. The total mass of Pvfps at each time interval after saline injection was determined by Bradford assay, revealing a fast initial secretion rate reaching a total mass of 50–60 μg after ca. 10 min (Fig. 1f). The similarity between artificial and natural secretions was further verified by performing MALDI-Tof MS analysis on a natural mussel's footprint deposited on glass. The MALDI peaks matched those from the saline-induced secretion, with Pvfp-3, -5 and -6 variants detected (Fig. 1e and Supplementary Table 1).

### Size and secondary structure of native Pvfps

**Figure 2:**
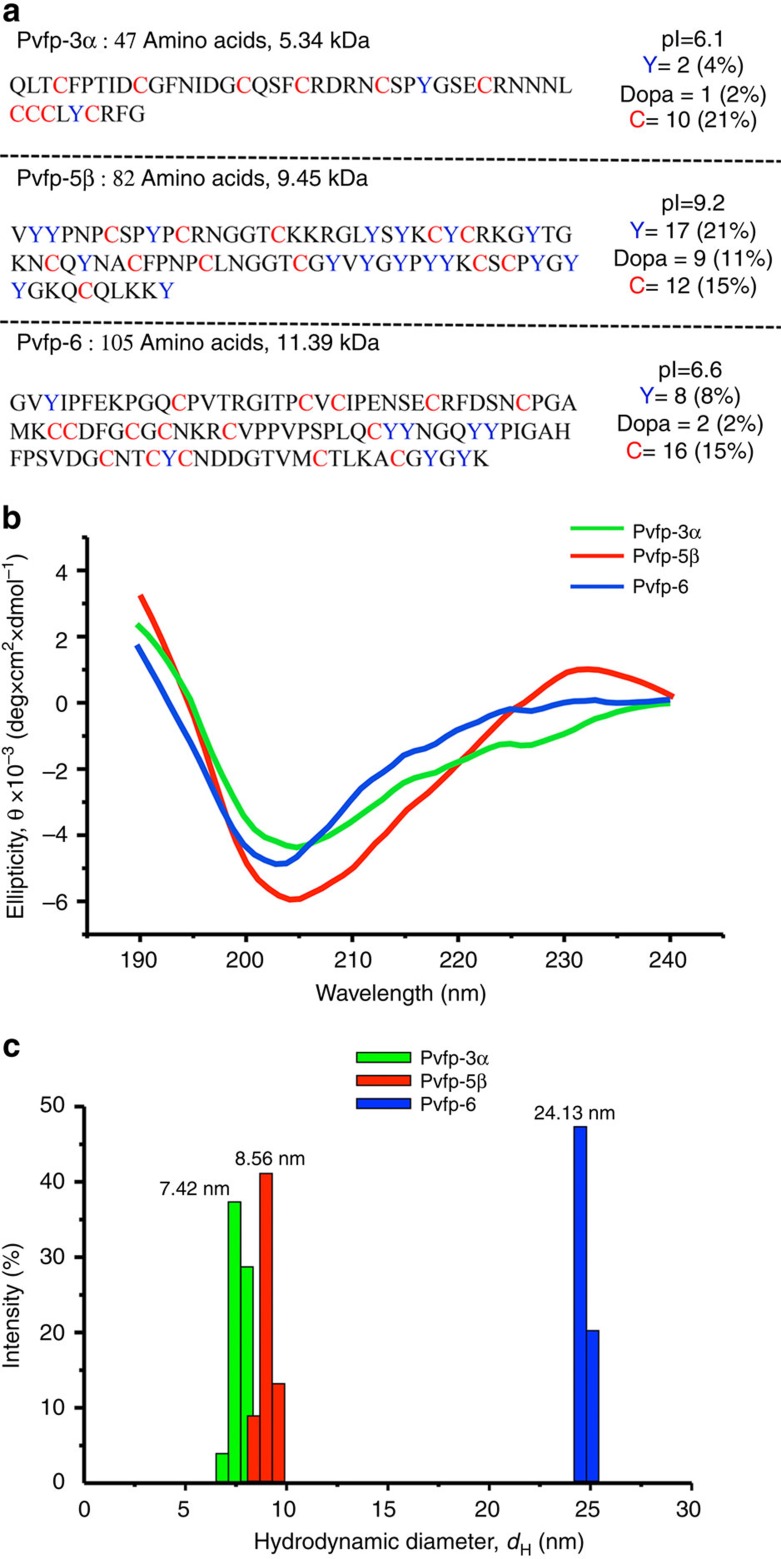
Native Pvfps possess largely random coil structures and form aggregates in solution. (**a**) Amino-acid sequences^22^ of Pvfps isolated from mussel foot and corresponding molecular weight, pI and content of Tyr (Y in blue), Dopa and Cys (C in red). (**b**) Circular dichroism spectra showing molar ellipticity (*θ*) as a function of wavelength, and (**c**) DLS hydrodynamic diameter (*d*_H_) distribution at 0.1 mg ml^−1^ in acid saline buffer at pH 4.

Pvfp-3α, -5β and -6 isolated and purified from mussel foot were shown to contain Dopa, as visible in the positive nitroblue tetrazolium (NBT) staining (Supplementary Figs 3 and 4), and their molecular weight was determined by MALDI-ToF MS (Supplementary Fig. 5). Pvfps are enriched with cysteine (Cys) and Tyr (Tyr)/Dopa residues, with Pvfp-3α possessing 10 Cys residues, accounting for 21 mol% of its amino-acid content, and Pvfp-5β having 21 mol% Tyr side chains in its primary sequence (Fig. 2a). Amino-acid analysis determined ca. 11 mol% Dopa content in Pvfp-5β (10 residues), whereas a lower content was detected for Pvfp-3α (1 residue; ∼2 mol%) and Pvfp-6 (2 residues; ∼2 mol%) (Supplementary Fig. 6). Circular dichroism spectra for all Pvfps exhibited predominantly random coil structures, with minima between 200 and 205 nm (Fig. 2b). The positive ellipticity for Pvfp-5β between 225 and 240 nm corresponds to π–π* transition of the aromatic side chains, that is, Tyr/Dopa^25^,^26^. Dynamic light scattering (DLS) revealed a hydrodynamic diameter of 7.42±0.44, 9.50±0.26 and 24.13±0.84 nm for Pvfp-3α, -5β and -6, respectively (Fig. 2c), indicating a different degree of aggregation in solution (Supplementary Note 1).

### Structural modelling of Pvfps

**Figure 3:**
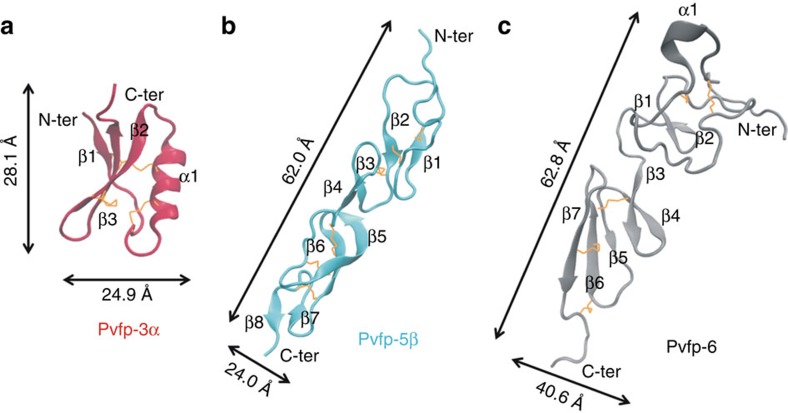
Molecular dynamics simulated structures of Pvfps display globular and elongated conformations. 3D homology models with average dimensions of (**a**) Pvfp-3α, (**b**) Pvfp-5β and (**c**) Pvfp-6 proteins with disulphide bonds shown in orange.

**Figure 4:**
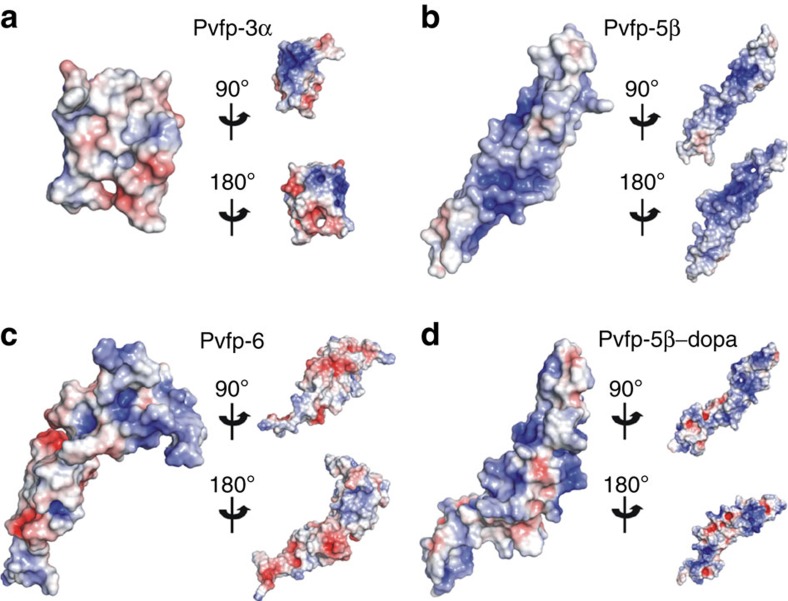
Electrostatic potential maps show differences in surface charge distribution among Pvfps. Electrostatic maps for (**a**) Pvfp-3α, (**b**) Pvfp-5β, (**c**) Pvfp-6 and (**d**) Pvfp-5β-Dopa at 90° and 180° rotation around their longitudinal axis. The surface potentials are represented from anionic (red) to cationic (blue) calculated at 0.25 M ionic concentration and 310 K.

To gain molecular-scale insights into the role of Pvfps on green mussel adhesion, we modelled the structure of Pvfp-3α, Pvfp-5β and Pvfp-6 by homology modelling and molecular dynamics simulations (see Methods for details). Pvfp-5β and Pvfp-6 adopt similar folds consisting of an extended, non-globular conformation. The structure of Pvfp-5β is predicted to contain a high percentage of β-strands (41% of the amino acids (aa)) forming anti-parallel β-sheets connected with loops (Fig. 3, Supplementary Figs 7 and 8). Pvfp-3α adopts a globular fold with a high percentage of β-strands (38% aa) and an α-helical region (aa 11–24). Pvfp-6 has a smaller percentage of β-strands (29% aa), a small α-helix (aa 15–19) and a higher percentage of loops. The Cys residues in Pvfp-5β are all oriented such as to engage in disulphide bridges. The α-helix in Pvfp-3α contains two cysteines, which are also predicted to form disulphide bonds with a Cys present in the C-terminal anti-parallel β-sheet region, thereby stabilizing the overall structure. Pvfp-5β shows a distribution largely of positive charges over its surface, with high abundance at the N-terminus region, and small patches of negative charges (Fig. 4b). In contrast, the surface electrostatic potentials for Pvfp-3α and Pvfp-6 display the presence of both negatively and positively charged regions (Fig. 4a,c). Such potentials are inevitably expected to be affected by micro-environmental changes, such as local pH and ionic strength. Given the established role of the post-translational modification of Tyr to Dopa in enhancing mussel adhesion, we modified all Tyr residues into Dopa (Pvfp-5β-Dopa). In comparison to Pvfp-5β simulations, the Tyr to Dopa modification does not reveal any significant structural change (Supplementary Fig. 9a–c), and only patches of negative potential appear on the Pvfp-5β-Dopa's surface because of the presence of an extra hydroxyl group on the Tyr residues (Fig. 4d).

**Figure 5:**
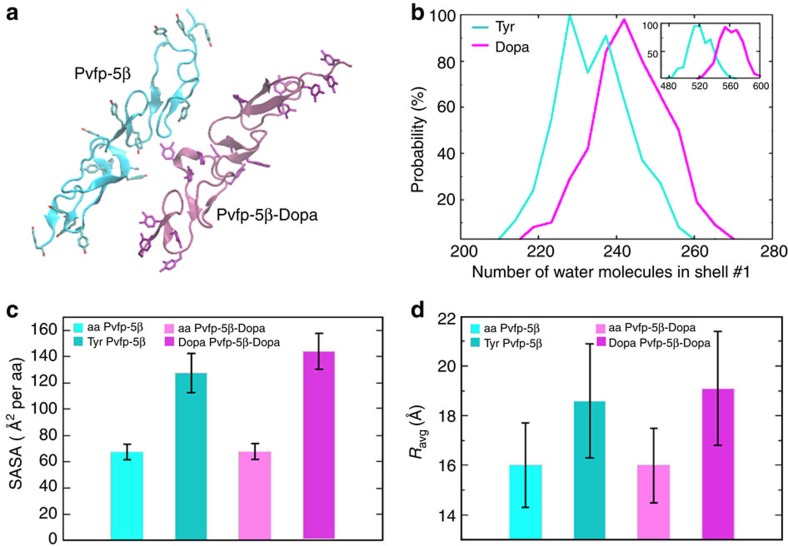
The modelled structure of Pvfp-5β-Dopa is more spread out and hydrated than Pvfp-5β. (**a**) Representative structure with Tyr (cyan) and Dopa (magenta) at the periphery of the protein. (**b**) Probability of water molecules in the first hydration shell around all Tyr and Dopa residues. Inset shows the same probability for all amino acids combined in Pvfp-5β and Pvfp-5β-Dopa. (**c**) Average SASA and (**d**) average distance from Tyr (dark cyan) or Dopa (dark magenta) to the protein centre (*R*_avg_) and for all other amino acids for Pvfp-5β (light cyan) and Pvfp-5β-Dopa (light magenta). Average SASA and *R*_avg_ values were calculated for the equilibrium structure and error bars represent s.d.

A detailed structural analysis of Pvfp-5β yields a conformational fold in which the Tyr residues are located at the periphery of the molecule with their aromatic rings facing the solvent (Fig. 5a). We found that for all Tyr within all conformations, the solvent accessible surface area (SASA) is 88% larger than that of other amino acids (127.5±14.9 versus 67.6±6.0 Å^2^; Fig. 5c). A similar trend was also observed for Pvfp-5β-Dopa, where the SASA was found to be further increased (144.3±13.6 versus 67.9±5.9 Å^2^ for other amino acids; Fig. 5c). The probability of water molecules in the first solvation shell within 3.4 Å of any atom of Tyr or Dopa showed an increase of 9% in the latter case (Fig. 5b), clearly suggesting that the 8% increase in solvation of Pvfp-5β-Dopa arises chiefly from the increased solvation of Dopa. We also computed the average distance of each amino acid to the protein centre and found that the average distance of all Tyr (18.6±2.3 Å) and Dopa (19.1±2.3 Å) is 16–19% higher than for other amino acids in the respective structures (Fig. 5d). Furthermore, the flexibility of the modelled Pvfp-5β, measured as the N- to C-terminus distance over the last 60 ns of the simulation varied from 72.07 Å (completely extended) to 49.43 Å (partially flexed; Supplementary Fig. 10 and Supplementary Movie 1).

### Adsorption studies of Pvfps on TiO_2_

**Figure 6:**
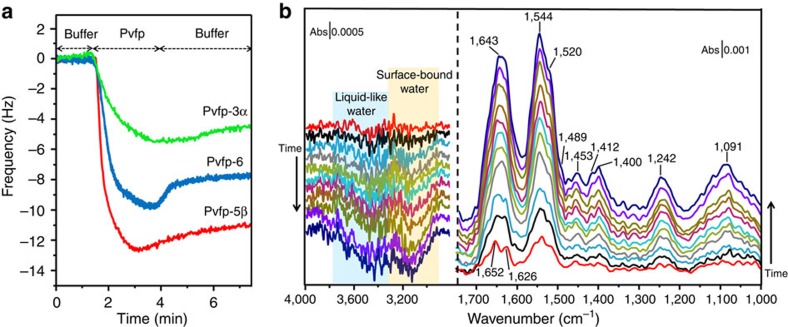
Pvfp-5β displaces surface-bound water from hydrated TiO_2_ and shows greater adsorption than other Pvfps. (**a**) QCM plots for the adsorption of Pvfp-3α, -5β and -6 on a TiO_2_-coated sensor. All proteins were adsorbed at a flow rate of 50 μl min^−1^ from a 0.1 mg ml^−1^ solution in acidic saline buffer. An initial equilibration period of ∼2 min was obtained by flowing the buffer, which was followed by the adsorption of the proteins (Pvfps), and finally by protein desorption under buffer flow. (**b**) ATR-IR spectra of adsorbed Pvfp-5β on TiO_2_ nanoparticle film from 0.1 mg ml^−1^ solution recorded at ∼2-min intervals. Background was from acid saline buffer on TiO_2_-coated ZnSe prism. The ∼3,300–2,900 cm^−1^ and ∼3,750–3,300 cm^−1^ infrared regions correspond to surface-bound (yellow shade) and liquid-like (blue shade) water, respectively.

To experimentally assess the adsorption behaviour of Pvfps, we conducted quartz crystal microbalance (QCM) and attenuated total reflection infrared (ATR-IR) spectroscopy measurements. QCM plots (frequency changes Δ*f* versus time) for the adsorption of Pvfps on TiO_2_ were obtained by initially flowing buffer to obtain a stable baseline (equilibration step, see Methods). In three separate experiments, 100 μl of 0.1 mg ml^−1^ Pvfps solutions were introduced for 2 min (adsorption step) before washing with buffer to desorb weakly bound Pvfps (desorption step). Pvfp-5β showed the greatest adsorption (Δ*f*_ads_=12.8 Hz), followed by Pvfp-6 (Δ*f*_ads_=9.7 Hz), and finally by Pvfp-3α (Δ*f*_ads_=5.4 Hz; Fig. 6a). The frequency shift normalized by the molecular weight, Δ*f*_ads_/molecular weight, is proportional to the number of protein molecules adsorbed on the QCM sensor. This value is highest for Pvfp-5β (1.32) and minimum for Pvfp-6 (0.86), showing that the latter protein has the least affinity for the TiO_2_ surface. The greater adsorption of Pvfp-5β on TiO_2_ was associated with the fastest initial adsorption kinetics, as evident from the slopes at the beginning of the adsorption curve. After washing, Pvfp-6 showed the greatest desorption (Δ*f*_des_=1.8 Hz), whereas Pvfp-5β and -3α desorbed in negligible amounts. The fast and abundant desorption of Pvfp-6 may be attributed to a sizeable fraction of its adhesive Dopa residues being screened within Pvfp-6 aggregates, which were observed from DLS, thereby reducing the availability of Dopa for surface binding.

In ATR-IR experiments, Pvfps were adsorbed from 0.1 mg ml^−1^ solution in acetic acid and 0.25 M KNO_3_ on a ∼100-nm thick TiO_2_ nanoparticle film. Pvfp-5β adsorbed strongly on TiO_2_ with intense absorptions recorded already in the first ATR-IR spectrum after 2 min (Fig. 6b). ATR-IR spectra of adsorbed Pvfp-5β revealed absorptions associated with peptide bonds, namely amide I, II and III at ∼1,640, 1,544 and 1,242 cm^−1^, respectively^27^. Other absorptions originating from CH_2_ aliphatic vibrations^28^ were observed between ∼1,460 and 1,400 cm^−1^. The broad band peaking at 1,091 cm^−1^ is generally associated with the bending mode (*δ*) of CH groups and *δ*(CH)+*δ*(OH) combination mode^29^. In addition, the peak at 1,520 cm^−1^ due to aromatic residues (Tyr/Dopa) was also observed, with a prominent shoulder at 1,489 cm^−1^ indicating Dopa/Ti(IV) coordinative bond^29^. The amide I band is particularly sensitive to protein's conformation and is routinely used for determinations of their secondary structure^27^. In the initial ATR-IR spectrum, this band exhibited two peaks at 1,652 and 1,626 cm^−1^, originating from random coils and β-sheets^27^, respectively, thus matching the modelled Pvfp-5β structure (Fig. 3b and Supplementary Fig. 8). The evolution of the amide I band shape can be attributed to structural rearrangement of adsorbed Pvfp-5β. In fact, the two initial well-resolved peaks merged over the course of the adsorption, indicating the emergence of a new contribution associated with β-sheets at 1,638 cm^−1^ and the concurrent loss of random coils at 1,655 cm^−1^, as revealed by the difference spectrum between the normalized initial and final ATR-IR spectra (Supplementary Fig. 11).

Another region of interest pertains to the stretching mode of OH groups (νOH) in the ∼3,800–2,800 cm^−1^ region, which primarily originates from water molecules involved in weak (liquid-like) and strong (surface-bound) hydrogen-bond interactions at higher and lower wavenumbers, respectively^30^. Negative bands progressively developed during the adsorption of Pvfp-5β on TiO_2_ (Fig. 6b) in the ∼3,800–3,300 cm^−1^ and ∼3,300–2,950 cm^−1^ regions due to dislodgment of liquid-like and surface-bound water molecules. Pvfp-5β thus exhibits the ability to displace interfacial water molecules from a wet hydrophilic surface (TiO_2_), subsequently enabling the formation of chemical bonds with the surface (inner-sphere complexes). In adsorption phenomena, outer-sphere complexes with solvent separation, that is, electrostatic interactions between a solvated anion and a surface metal cation, can be the precursor of an inner-sphere complex, such as metal–ligand coordination bonds^3^. The presence of coordination bond between catechols (such as Dopa) and Ti(IV) is characterized by a doublet at *ca.* 1,490 and *ca.* 1,270 cm^−1^ attributed to ν(CC) of aromatic rings and ν(CO) modes, respectively^29^,^31^. The negative second derivative of the ATR-IR spectra (Supplementary Fig. 12) confirmed the presence of a doublet at 1,482 and 1,270 cm^−1^ in the initial spectra. The former peak gradually disappeared as the adsorption progresses and the TiO_2_ surface became saturated, indicating that Dopa side chains coordinated Ti(IV) leading to structural rearrangements and eventually to an enrichment of β-sheet in the adsorbed protein layer. Pvfp-3α and -6, in contrast, were not able to adsorb significantly on TiO_2_, perhaps because of their lower Dopa content as compared with Pvfp-5β. Removal of interfacial water is well recognized to be a major challenge in underwater adhesion^1^, and the presence of Dopa appears to be critical for enabling this behaviour.

### Surface adhesion of adsorbed Pvfps layers

**Figure 7:**
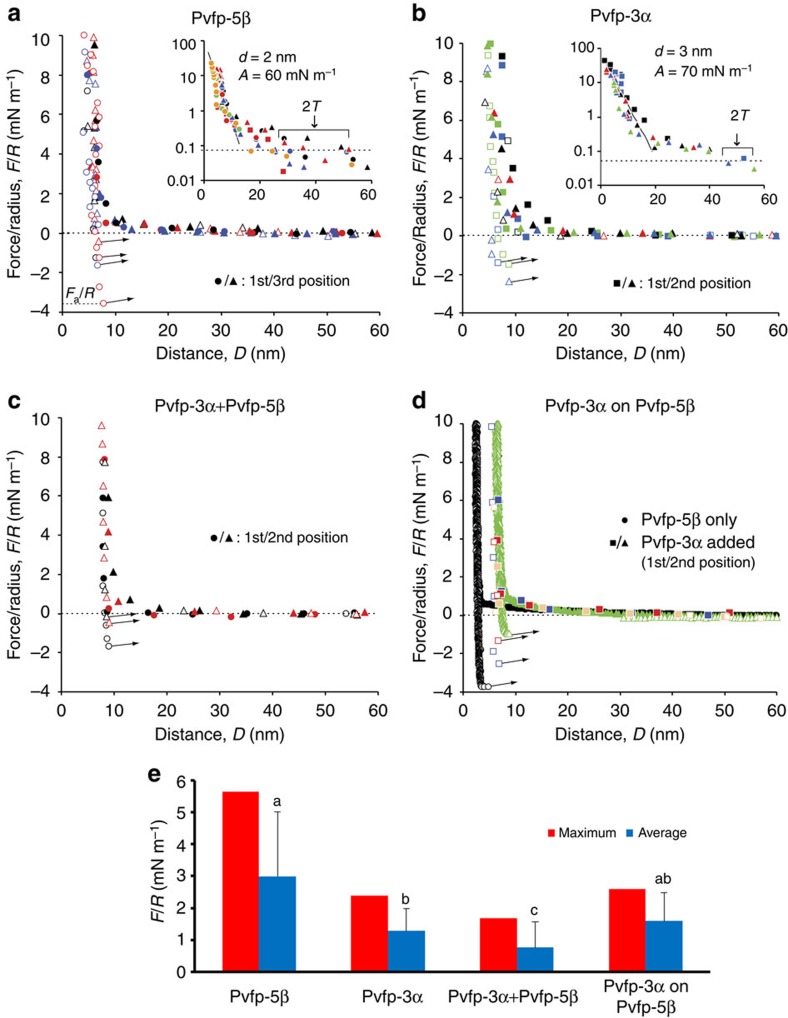
Pvfp-5β has greater surface adhesion than Pvfp-3α and their mixture. (**a**,**b**) Normalized force *F/R* measured with the SFA as function of the surface separation distance *D* after adsorption of Pvfp-5β or Pvfp-3α on mica in saline buffer with pH 3. The insets show the range of repulsion 2*T*, force detection threshold (dotted line) and exponential curve *F*/*R*=*A*e^−*D/d*^. (**c**) Force measured after simultaneously adsorbing Pvfp-3α and Pvfp-5β from a mixed solution in acid saline buffer with pH 4. (**d**) Force measured after adsorption of Pvfp-5β on mica (black data points) and after adsorbing Pvfp-3α on Pvfp-5β (coloured points) from the same acid saline buffer with pH 4. Each symbol corresponds to one contact position and different colours indicate different surface approach/retraction cycles, with filled/open symbols indicating approach/retraction, respectively. The surfaces jumped out from adhesive contact at points indicated with arrows, where the adhesive force reached the minimum *F*_a_*/R*. (**e**) Maximum and average adhesion force (*F*/*R*) performed in symmetric mode for Pvfp-5β, Pvfp-3α, Pvfp-3α+Pvfp-5β equimolar mixture, and Pvfp-3α on Pvfp-5β. Results of Welch's *t*-test comparisons are presented. Means that do not share a letter are significantly different.

The adhesion capability of Pvfps was assessed by surface force apparatus (SFA) experiments, using procedures established for *Mytilus* mussel adhesive proteins^16^,^17^,^18^. When two mica surfaces were coated with layers of Pvfp-5β and brought into contact in acid saline buffer, the initial force *F* measured on separating the surfaces was adhesive (Fig. 7a). The maximum adhesion measured in four independent experiments (with different pairs of mica surfaces) was *F*_a_*/R*=5.6 mN m^−1^, corresponding to a work of adhesion^32^ *W=*(2/3*π*)*F*_a_*/R*=1.2 mJ m^−2^. Adhesion decreased with time as the surfaces were repeatedly approached and retracted at a contact position, and as different positions were tested (compare first and third positions in Fig. 7a and Supplementary Fig. 13), most likely due to Dopa oxidation^15^. The average value (±sample s.d.) of the adhesive force was *F*_a_*/R*=3.0±2.0 mN m^−1^. The force was purely repulsive during surface approach, showing that adhesion was generated by the bonds created by Pvfp-5β molecules during surface contact. The force consistently exceeded the detection threshold when the surface separation distance *D* became smaller than 2*T*=25–50 nm (Fig. 7a, inset). Since the Debye length of the saline buffer was smaller than 1 nm (ref. 32), such long repulsion range 2*T* can be attributed to the overlap between protein layers adsorbed on opposite surfaces, each having an approximate thickness *T*=12–25 nm. This value is larger than the size of a Pvfp-5β molecule (Fig. 3b), and corroborates the DLS result (Fig. 2c) that Pvfp-5β forms aggregates, most likely via Dopa–Dopa crosslinks. The largest values of *T* were obtained on approaching the surfaces for the first time at a given contact position, indicating that adsorbed molecules and aggregates could be irreversibly compacted or flattened on the substrate, perhaps due to the formation of additional Dopa–Dopa covalent crosslinks or Dopa–mica hydrogen bonds on compression. The force curve became approximately exponential at distances *D*<15 nm, with a decay length of about 2 nm up to the maximum load considered *F/R*≈20 mN m^−1^ (Fig. 7a, inset). The same curve was obtained for multiple approaches at the same contact position. This behaviour suggests that Pvfp-5β was adsorbed as a soft hydrated layer, as opposed to a compact ‘hard-wall' coating, allowing water and protein molecules to move and rearrange as the compressive force was increased.

The force curves obtained after adsorbing Pvfp-3α on the mica surfaces of the SFA (Fig. 7b) showed a repulsion range 2*T*≈50 nm and exponential regime at small distances, with *d=*3 nm and *A=*70 mN m^−1^, similar to those obtained for Pvfp-5β. On the other hand, the adhesion force was significantly weaker: the maximum value measured in three independent experiments was *F*_a_*/R*=2.38 mN m^−1^ and the average *F*_a_*/R*=1.3±0.7 mN m^−1^. Analysis of variance of the adhesion force data for Pvfp-3α (11 measurements) and Pvfp-5β (9 measurements) using Welch's *t*-test showed that the difference was statistically significant, with probability *P*<4% to be accidental (Fig. 7e). This result is in line with QCM and ATR-IR adsorption measurements, showing that Pvfp-5β exhibits a more surface adhesive character than Pvfp-3α. Since Pvfp-5β is the first adhesive protein to be secreted after stimulating the mussel foot with saline injection (Fig. 1), it is interesting to study how Pvfp-5β interacts with Pvfp-3α. When both Pvfp-3α and Pvfp-5β were simultaneously adsorbed from an equimolar mixed solution, the maximum adhesion force was *F*_a_*/R*=1.7 mN m^−1^, smaller than the values measured for either Pvfp-5β or Pvfp-3α alone (Fig. 7c). Also the average value (0.8±0.8 mN m^−1^) showed a significant decrease compared with Pvfp-5β (*P*<3% for two independent experiments and three different measurements). We hypothesize that the observed delay in secretion between Pvfp-3 and Pvfp-5 may help avoid such interference between Pvfp-3 and the adhesive residues of Pvfp-5. When Pvfp-5β was first adsorbed and followed by Pvfp-3α, the maximum adhesion force decreased to *F*_a_*/R*=2.6 mN m^−1^, close to the value measured for Pvfp-3α alone (Fig. 7d). Therefore, Pvfp-3α appeared to almost completely screen and ‘replace' the adhesive properties of the Pvfp-5β layer, although statistical analysis indicates that the lower adhesion observed after adsorbing Pvfp-3α on Pvfp-5β compared with Pvfp-3α or Pvfp-5β alone may as well be due to random experimental variations. These findings support the idea that time-regulated secretion of Pvfps is designed at creating a functional multilayer at the interface between the adhesive plaque and the target surface for mussel attachment. In this multilayer configuration, Pvfp-5 is the main adhesive or primer in direct contact with the surface, whereas Pvfp-3 provides the first functional layer for the attachment of other proteins, such as Pvfp-6.

## Discussion

Our understanding of mussel adhesion has broadened in recent years beyond the unambiguous role of Dopa chemistry in both marine^1^ and freshwater mussels^5^,^33^, revealing that more intricate sequence features of mussel adhesive proteins are at play in enabling wet-resistant adhesion^17^,^18^,^19^. This study further unveils critical time-dependant and molecular-scale mechanisms of mussel adhesive proteins, using the Asian green mussel as a model system. On triggering the process of byssus formation, Pvfp-5 is consistently the first protein to be secreted, followed by Pvfp-3, and ultimately Pvfp-6. These findings are distinct from those observed for *Mytilus*^16^, in which Dopa-rich mfp-3 is initially detected from the mussel foot, followed by mfp-6. A plausible explanation for the non-detection of the other plaque protein mfp-5 is perhaps related to the high laser power needed to desorb it from the plaque footprint by MALDI-ToF MS.

It should be noted that upon saline injection, Pvfps are secreted over a longer timescale (*ca.* 15 min) compared with the natural secretion (*ca.* 1 min) (Supplementary Fig. 14 and Supplementary Movie 2). KCl solution injection into the mussel pedal ganglion increases the concentration of K^+^ ions ([K^+^]) outside nerve cell membranes, disrupting the K/Na steady state resting balance and, consequently, the resting potential (depolarization)^34^,^35^. The effect of the increase of [K^+^] on a cell membrane potential has been shown for a frog muscle fibre: the membrane potential rapidly increased, and only a small repolarization occurred as [K^+^] returned to normal, followed by rectification with Cl^−^ ions^36^,^37^. Similarly, the saline injection depolarizes the mussel pedal ganglion, turning on all foot processes. Unlike the well-coordinated on–off process of natural thread formation, the saline injection causes a sustained ‘on' process that lingers until ion balance has been restored (J.H. Waite, personal communication). It is important to notice that the saline-induced secretion does nonetheless lead to the formation of a functional adhesive byssus (Fig. 1b–d).

Three *P. viridis* adhesive proteins isolated from foot glands were re-dissolved in acidic solution at pH 4, which is consistent with the acidic pH imposed by marine mussels during plaque formation^16^,^38^. Pvfps display a predominantly random coil structure and form aggregates, with Pvfp-6 aggregates containing significantly more protein molecules than Pvfp-3α and -5β aggregates (Supplementary Note 1). The 3D modelled structure of Pvfp-5β and Pvfp-6 adopt an extended, non-globular conformation with high aspect ratio, whereas Pvfp-3α is more globular. In agreement with circular dichroism (Fig. 2b) and ATR-IR (Fig. 6b) spectroscopic analyses, all Pvfps are largely unstructured (that is, high content of random coils), with Pvfp-5β having the highest percentage of anti-parallel β-sheets (ca. 40%) connected by loops (ca. 60%). Molecular dynamics simulations revealed that all Tyr residues in Pvfp-5β are located on the protein surface and that they are 88% more exposed to the solvent than the other amino acids. When each Tyr in Pvfp-5β was converted into Dopa, the resulting molecular dynamics simulations showed a further increase (9%) in the number of water molecules solvating Dopa side chains compared with Tyr.

Having >35% random coils, Pvfp-5β is considered highly unstructured^39^, which parallels the predominantly unordered structure of *Mytilus* mfp-3 (refs 17, 25, 40) as measured by circular dichroism. In previous molecular dynamics simulations, Qin and Buehler^41^ assumed disordered structures for mfp-3 and -5 with exposed Tyr residues, although no homology modelling was carried out in this study. Using these assumptions, Tyr SASA values in these proteins (78.8±15.0 and 109.3±12.7 Å^2^ per amino acid for mfp-5 and -3, respectively) were significantly lower than those obtained for Pvfp-5β (127.5±14.9 Å^2^), which further increased when all Tyr are converted into Dopa (144.3±13.6 Å^2^). Furthermore, *R*_avg_ of Pvfp-5β comprising all Tyr and all Dopa residues is 18.6±2.3 and 19.1±2.3 Å, respectively (16–19% higher than other residues), whereas those for the assumed disordered structures of mfp-3 and -5 were more compact with *R*_avg_ of 11.0 and 9.3 Å, respectively (4–10% larger than other residues). Our homology modelling simulations thus allow us to conclude that Tyr/Dopa residues of mussel adhesive proteins are considerably more exposed to the solvent than when assuming purely disordered structures^17^,^25^,^41^, with Pvfp-5β adopting a less-folded structure with a higher aspect ratio. Pvfp-5β conformation with all Tyr exposed to the solvent appears as a functional adaptation (i) to facilitate specific interactions with tyrosinase, the enzyme involved in the posttranslational modification of Tyr into Dopa; and (ii) to maximize the density of Dopa at the protein surface. The modelled random coil N-terminal domain (amino acids 1–15), in particular, lacks a stable, specific 3D structure that along with the protein's intrinsic flexibility at its centre (extended to partially flexed transition; Supplementary Movie 1 and Supplementary Fig. 10) provide structural plasticity to adapt the plaque protein to varying conformational requirements, in particular for surface interactions^39^,^42^,^43^.

Adsorption studies of native Pvfps conducted on TiO_2_, a model surface frequently chosen to mimic metal oxide and mineral surfaces encountered by fouling organisms, reveal a clear ranking of adsorption. Pvfp-5β showed the highest adsorption in QCM assays, followed by Pvfp-3α and -6. ATR-IR spectroscopy detected considerable spectral intensities from adsorbed Pvfp-5β binding to Ti atoms via coordination bonds. Pvfp-5β was able to displace liquid-like and surface-bound water molecules from TiO_2_, thus overcoming repulsive hydration forces and enabling the formation of inner-sphere complexes. Pvfp-6 and -3α were not detected, demonstrating their inability to strongly adsorb on wet hydrophilic surfaces. The characteristic doublet of Dopa/Ti bidentate coordination gradually disappeared over time, as surface available sites became saturated. In agreement with the protein's modelled structure, the initial ATR-IR spectrum of adsorbed Pvfp-5β showed a predominance of β-sheets and random coils, with the latter partially converting into β-sheets over time (Supplementary Fig. 11). Upon adsorption, the unordered conformation needs not to remain so, and an ordered structure can arise (that is, β-sheet) to stabilize the adsorbed structure. The hydrophobic mfp-3S has been shown by Overhauser dynamic nuclear polarization relaxometry to be the only mfp able to evict hydration layers from a hydrophobic surface (polystyrene), although no mfp was able to displace water molecules from a hydrophilic surface (silica)^44^. *In situ* surface dehydration studies of mussel adhesive proteins are challenging to carry out in laboratory conditions that mimic closely the natural environment. To this end, SFA and Overhauser dynamic nuclear polarization experiments pose difficulties. The former is performed under applied compressive forces and the latter requires a surface to be spin labelled with nitroxide radicals. On the other hand, ATR-IR spectroscopy used in this work is an *in situ*, label-free technique that allows unperturbed investigations of mussel adhesive proteins interacting with hydrated surfaces.

SFA measurements complement the picture gained from QCM and ATR-IR adsorption studies, with Pvfp-5β exhibiting the largest adhesive force when coated between mica surfaces, that is, an adhesion force of 3.0–5.5 mN m^−1^, corresponding to a work of adhesion of 0.6–1.1 mJ m^−2^. These values are in close agreement with those reported for the adhesion of Dopa-rich mfps adsorbed on mica under similar conditions of pH, salinity, protein concentration and adsorption time^1^. In contrast, Pvfp-3α showed a weaker adhesion force (1.5–2.4 mN m^−1^), in line with previous SFA studies on mfps^1^. Notably, the co-adsorption of Pvfp-5β and Pvfp-3α resulted in an adhesion force of 1.7 mN m^−1^, which is weaker than that of Pvfp-5β and Pvfp-3α alone. In other words, the co-secretion of these two proteins in the early phase of mussel attachment would be detrimental for adhesion. Taken together, our adsorption and SFA measurements thus quantitatively explain the stepwise secretion in mussel adhesive proteins. Very recently, Maier *et al*.^23^ have revealed by SFA the synergistic interplay between Dopa and lysine (Lys) in the bioadhesion of siderophores, a type of bacterial iron chelators. Adjacent Dopa–Lys residues promote adhesion in seawater, whereby Lys initially displaces hydrated surface cations and facilitates the subsequent Dopa binding to underlying substrates. Interestingly, Pvfp-5β also comprises Lys in proximity to Dopa residues (three adjacent pairs and five pairs separated by one amino acid; Fig. 2a and Supplementary Fig. 15). On the other hand, Pvfp-3α and Pvfp-6 have no Lys and only one adjacent Dopa–Lys pair, respectively. Together with the efficient water displacement capability and superior adsorption of Pvfp-5β, these sequence distinctions are in full agreement with the recent discovery that Dopa–Lys pairs are critical in enhancing underwater adhesion.

**Figure 8:**
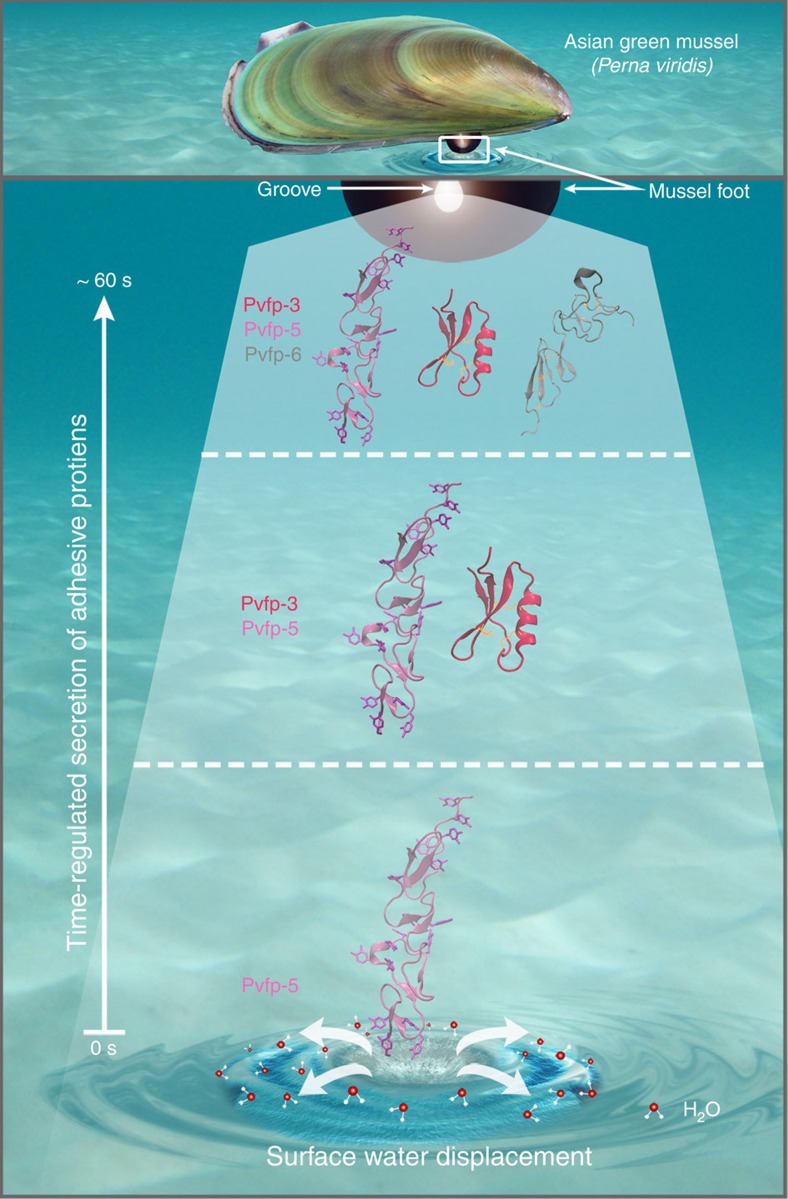
Mussel wet adhesion is dictated by time-regulated secretion of adhesive proteins. The Asian green mussel (on top) secretes adhesive proteins (Pvfp-3, -5 and -6) from the groove located at the tip of its foot. Pvfp-5 is the first protein to be secreted and is able to displace surface-bound water molecules from wet surfaces. The modelled structure of Pvfp-5 indicates that all Dopa/Tyr residues are strategically located on the periphery of the elongated protein to maximize the interactions with surfaces and other adhesive components. Furthermore, Pvfp-5 contains multiple pairs of Dopa–Lys located in close proximity from each other (Supplementary Fig. 15), which further contributes to surface water displacement. Pvfp-3 is subsequently secreted in addition to Pvfp-5, followed by the addition of Pvfp-6 in the last step of plaque deposition.

Considering the growing evidence that adhesive proteins in mussels and other fouling organisms are secreted as a complex fluid, that is, coacervate^19^,^45^,^46^, an interesting question will concern the effect of homogenous solutions on adhesive proteins' interactions and structures. Notwithstanding this complexity, a clear model of wet bioadhesion in mussels of *P. viridis* emerges from our findings, whereby a Dopa-rich primer (Pvfp-5) is initially secreted because of its superior adsorptive and adhesive abilities, with a characteristic spread-out conformation to maximize interactions with foreign surfaces. The primer's ability to displace surface-bound water from hydrophilic surfaces allows the formation of strong and durable bonds via its adhesive Dopa residues exposed on the protein surface, hence creating an environment conducive to the assembly of other plaque components (Fig. 8). The subsequent secretion of a Cys-rich protein (Pvfp-3) is likely to protect Dopa residues of Pvfp-5 from oxidation, through the formation of cysteinyl–Dopa bonds and disulphide bridges within the adhesive plaque, as observed for mfps. Given the high content of Cys within Pvfp-5β (15 mol%), another plausible scenario is that intramolecular disulphide bond formation could also protect Dopa residues from oxidation via redox chemistry. In other words, Pvfp-5β may be able to auto-protect itself against Dopa oxidation (and thereby minimizes loss of adhesion), a hypothesis that will be tested in future work.

## Methods

### Time-resolved protein secretion

The adhesive protein secretion in mussels of *P. viridis* was triggered by 1-ml injection of 0.56 M KCl, phosphate buffered at pH 7.2 into the mussel's pedal nerve located at the base of the foot^16^,^47^,^48^. This process was demonstrated to mimic the mussel's natural secretion, thus being virtually indistinguishable^48^,^49^. Proteins exuded were collected from the distal depression of the mussel foot, the known location of plaque and adhesive protein secretion, by swabbing with a pipette tip, which was rinsed with 10 μl of 5% acetic acid. A total of *n*=15 mussels were considered. Proteins were collected before saline injection and at a specific time intervals after injection: 10 s, 30 s, 1, 3, 5, 10 and 30 min. The obtained fractions were analysed by MALDI-ToF MS by spotting 1.5 μl of protein solution sample on the MALDI plate and applying 1.5 μl sinapinic acid (Sigma-Aldrich) as sample matrix. Quantification of the total protein concentration collected at each time interval from the saline-induced mussel secretion was determined spectrophotometrically by Bradford assay. A series of standard bovine serum albumin (BSA) concentrations ranging from 0 to 2,000 mg ml^−1^ (0, 25, 125, 250, 500, 750, 1,000, 1,500 and 2,000 μg ml^−1^) were prepared by diluting an ampule of albumin standard at 2 mg ml^−1^ in 0.9% aqueous NaCl solution containing sodium azide (Thermo Scientific) with 5% acetic acid. The assay was performed by mixing 250 μl of the Coomassie reagent solution (Coomassie Bradford Protein Assay kit; Thermo Scientific), 5 μl of the BSA standard and each protein sample into a microplate (Nunclon 96 Flat Transparent, NUNCTM). The mixtures were allowed to incubate for 10 min at room temperature. The absorbance of each mixture was measured at 595 nm with a plate reader (Infinite M200 Pro; TECAN). The protein concentration of the unknown samples was calculated from the equation obtained from the quadratic best-fit curve of the BSA concentration standard.

### Isolation and purification of Pvfps

Pvfps were extracted from the mussel's foot organ of *P. viridis* mussels collected from the northern coast of Singapore (Johor Strait 1.2643°N, 103.4226°E). Their phenolic glands were dissected and stored at −80 °C. Once frozen, the pigments on 50 glands were scraped off and the glands were homogenized in 150 ml of 5% acetic acid (Schedelco), containing protease inhibitors, pepstatin and leupeptine (Thermo Scientific). The homogenate was centrifuged (S3033-High Speed Centrifuge J-26XP) at 20,000*g* at 4 °C for 40 min. The supernatant was then collected for further purification by high-performance liquid chromatography (HPLC) (Agilent 1,260 Infinity, USA). The protein was first purified using a size-exclusion column (Agilent Bio SEC-5 columns, 150 Å) with a 5% acetic acid mobile phase at 1 ml min^−1^, which resulted in two major peaks after about 5 and 10 min detected at both 220 and 280 nm (Supplementary Fig. 3). The fractions of the peak B were pooled and ran through a reversed phase column (Agilent ZORBAX 300SB-C8). The proteins were eluted with an aqueous acetonitrile (Fulltime) gradient containing 0.1% trifluoroacetic acid (TFA) (Sigma-Aldrich) at 2 ml min^−1^ (Supplementary Fig. 4). Acid urea polyacrylamide gel electrophoresis with 10% acrylamide was performed to assess the purity of the protein fractions. A 1:1 ratio of protein fraction and loading dye (0.15 mM methyl green, 4 M urea and 5% acetic acid) were prepared. Gels were pre-equilibrated with the loading dye (10 μl per well) with a 5% acetic acid tank buffer at a constant current of 40 mA. Subsequently, a total volume of 10 μl of the prepared samples was loaded in each well. The gels were then run at a constant current of 40 mA. Two sets of gels were prepared for each sample to detect protein bands by staining with Coomassie Blue R-250 (Applichem; 0.125% Coomassie Blue R-250, 50% methanol, 10% acetic acid), and redox-cycling proteins by NBT (Roche) (40 μl NBT in 50 ml of 2 M sodium glycinate at pH 10). These gels were then de-stained with 7% acetic acid, 5% methanol and sodium borate solution. The molecular weights of the purified proteins were identified by MALDI-ToF MS spectrometer (Axima-TOF2TM, Shimadzu Biotech). A 1:1 ratio of sinapinic acid matrix (Sigma-Aldrich; 20 mg ml^−1^ and 1 μl) and the samples resuspended in 5% acetic acid (1 μl) were vortexed and loaded onto a MALDI plate. The samples were air dried and inserted into the instrument for analysis.

### Amino-acid analysis

The presence and amount of Dopa side chains in Pvfps were determined by amino-acid analysis. Up to 30 μl of each protein were hydrolysed under vacuum at 110 °C with 150 μl of 6 M HCl (Fluka) and 10 μl phenol (Sigma) at 5% w/v for 8 h. The hydrolysates were flash evaporated using a speed vacuum (Scan Speed, USA), washing them twice with water and methanol. The proteins were then resuspended in 300 μl of sample dilution buffer (SYKAM, Germany). The samples were centrifuged at 14 r.p.m. for 5 min. The supernatants were transferred into glass vials and loaded into an amino-acid analyser (SYKAM Amino Acid Analyzer S433, Germany). Amino-acid standard (SKYAM, Germany) and a Dopa standard (Sigma) at 1 mg ml^−1^ dissolved in the sample dilution buffer were loaded. The amount of Dopa was then quantified by correlating the amino-acid composition chromatogram of the Pvfp samples with that of the standards.

### Circular dichroism

Pvfps were dissolved in 10 mM acetic acid at a concentration of 1 mg ml^−1^, and their secondary structure was determined by circular dichroism using a Chriascan spectropolarimeter (Model 420, AVIV Biomedical Inc.). Measurements were conducted in triplicate at wavelengths ranging from 190 to 260 nm, with 1 nm step size and 1 nm bandwidth. The spectra were smoothed by the Savitzky–Golay method with a polynomial order of 2.

### Dynamic light scattering

The hydrodynamic diameters of Pvfps were determined by DLS. Proteins were solubilized in 10 mM acetic acid at 0.1 mg ml^−1^ and analysed with a 90Plus particle size analyser (Brookhaven Instruments) equipped with a 658.0-nm monochromatic laser. To minimize the reflection effect, all measurements were taken at a scattering angle of 90°. The number weighted histogram profiles of the proteins in solution were used for data analysis.

### Homology modelling

There are no crystal structures available for the Pvfps, so their 3D structures were generated using standard protein homology modelling methods. The full-length sequence of Pvfps-3α, -5β and -6 with 47, 82 and 105 residues, respectively^20^, was used as query to retrieve structural templates from the protein databank database using BLASTp^50^. This method searches for sequences that are homologous to the query sequence. Only Pvfp-5β yielded two homologous structures with sequence identity >30%, namely DEL-1 EGF domain (PDB ID: 4D90, resolved at 2.60 Å (ref. 51)), a cell adhesion protein with 51% sequence identity, and Human NOTCH-1 EGFS domain (PDB ID: 2VJ3, resolved at 2.60 Å (ref. 52)), involved in the NOTCH signalling pathway, with 39% sequence identity. Like Pvfp-5β, both proteins are disulphide rich. These two proteins were then used as templates to build a 3D model of Pvfp-5β using Modeller^53^, a programme based on comparative protein structure modelling by satisfaction of spatial restraints to generate 10 models. Superimposition of all 10 models of Pvfp-5β generated using Modeller revealed conserved secondary structures with some differences in the conformations of the flexible loop regions. One structure was selected for our studies based on the lowest discrete optimized protein energy (DOPE) score^54^, which ranged from −5,580 to −5,135. In the case of Pvfp-3α and Pvfp-6, no template structure was identified with sequence identity >30%. To generate the 3D models of these two proteins, the automated I-TASSER pipeline^55^, which is based on multi-threading alignment and iterative template fragment assembly simulation, was used. For consistency, a 3D model of Pvfp-5β was also generated using I-TASSER^56^ and was found to be similar to that generated using Modeller. The Modeller-generated Pvfp-5β model with the lowest DOPE score, and the Pvfp-3α and Pvfp-6 models with low *C*-scores^56^ and high structural similarity to Pvfp-5β were chosen to be used as starting models for initiating molecular dynamics simulations for additional refinement. Although the structures are composed of many flexible regions (Supplementary Fig. 7b–d), visual inspection of the trajectories revealed that the predicted disulphide bonds stabilize the structure as well as maintain the tertiary shape of the protein.

### Molecular dynamics simulations

Simulations were performed using the Amber12 (ref. 57) ff99SB force field^58^. For simulations of Pvfp-5β modified with Dopa, the partial charges and the force-field parameters were generated using the *Antechamber* module in AMBER. The *Xleap* module of Amber was used to prepare the system by adding hydrogen atoms. The structures were solvated using a box of TIP3P water molecules^59^. An octahedral box was constructed such that a minimum distance of 10 Å was ensured between any atom of the protein and the boundary of the box. An appropriate number of Na^+^ and Cl^−^ counter ions were further added to each system, to ensure net charge neutrality. All short-range, non-bonded van der Waals interactions were truncated at 9 Å and the particle mesh Ewald method^60^ was used to model the long-range electrostatic interactions. The SHAKE algorithm^61^ was used to constrain bond vibrations involving hydrogen atoms. Solvent water molecules and counter ions were initially relaxed by energy minimization, keeping the protein atoms restrained. This was followed by relaxing the entire system using energy minimization to relieve any steric clashes. Each system was next subjected to molecular dynamics simulations using the following protocol: an equilibration phase where harmonic restraints were placed on the Cα atom with a force constant of 25 kcal mol^−1^ Å^−2^ and the system gradually heated from 50 to 300 K for a period of 250 ps. This was followed by a gradual reduction of the restraints over the next 250 ps until the restraints were reduced to 0; the system was allowed to evolve at 300 K over the next 2,000 ps without restraints. Finally, the equilibrated structures were subjected to three independent (different initial velocities) 100-ns molecular dynamics simulations (totalling 300 ns for each system), and conformations were saved every 10 ps. Analysis of the simulations were carried out using the *ptraj* module. Before the analysis, the conformations generated in molecular dynamics simulations were centred and root mean square (r.m.s.) fitted to remove overall translation/rotations. The conformations sampled during the simulations for all the three models were stable as judged by the temporal evolution of r.m.s. deviation of Cα atoms. The simulations reached an equilibrium state within ∼40 ns in all the simulations (Supplementary Fig. 7a), and therefore the analysis was carried out over the last 50 ns. Residue accessible surface area was calculated using Naccess V2.1.1 (ref. 62). The SASA, over the trajectory for each amino acid was computed by assigning each atom an average radius of 2 Å and by summing up the total surface area. The trajectories were viewed in VMD^63^ and the images were created in VMD and PyMol^64^. The electrostatics calculations for each protein were carried out at 0.25 M ion concentration to replicate experimental conditions using the APBS plugin^65^ available in PyMol.

### Quartz crystal microbalance

The 5-MHz piezoelectric quartz crystals coated with TiO_2_ were purchased from Q-Sense, Sweden. The crystals were ozone treated for 10 min prior and after immersion in 2% SDS in purified water (Milli-Q, Millipore) for 30 min. The cleaned surfaces were then washed thoroughly with purified water, dried with nitrogen gas and used immediately. Real-time QCM measurements were performed using a Q-Sense E4 QCM (Q-Sense, Sweden) instrument with four-channel IPC pump (Ismatec SA, Switzerland). Three cleaned crystals were mounted in the micro-fluidic chambers of the QCM. On attaining stable resonant frequencies, 0.1 mg ml^−1^ of each protein was injected in the three channels, respectively, at a constant flow rate of 50 μl min^−1^. Pvfps were prepared in degassed 10 mM acetic acid at a concentration of 0.1 mg ml^−1^. All experiments were repeated at least three times, with a s.d. of the resulting frequency change of <3% for the proteins used in the QCM adsorption experiments.

### Attenuated total reflection infrared spectroscopy

ATR-IR spectra were recorded with a MIRacle accessory equipped with a three-reflection ZnSe prism (Pike Technologies) in an infrared spectrometer (Vertex 70, Bruker) equipped with a KBr beamsplitter and a liquid nitrogen-cooled mercury cadmium telluride detector. The optical bench was continuously purged with nitrogen gas. The ZnSe ATR prism was coated with a ∼100-nm thin film of TiO_2_ (anatase; <25 nm in diameter) (Sigma-Aldrich) nanoparticles by drying 50 μl of 10^−3^ M sonicated aqueous TiO_2_ suspensions overnight in air^66^,^67^,^68^. A volume of 40 μl of Pvfps at 0.1 mg ml^−1^ dissolved in 10 mM acetic acid and 0.25 M KNO_3_ was placed on the TiO_2_ nanoparticle film-coated ZnSe prism, and ATR-IR spectra were collected from 128 co-added scans at 4 cm^−1^ resolution with Opus software (version 6.5). Background was from 10 mM acetic acid and 0.25 M KNO_3_ on the TiO_2_ film. The spectra were corrected for the frequency dependence of the penetration depth with the ATR correction programme available in the Opus 6.5 software.

### Surface forces apparatus

Adhesion and normal force measurements between mica surfaces bearing adsorbed layers of Pvfps were obtained using a SFA (Mark III from Surforce LLC, USA). A detailed description of the technique can be found in Israelachvili and McGuiggan^69^. Atomically smooth mica sheet were coated with a thin Ag layer on one side, glued on cylindrical glass supports with radius *R*=1.8–2.2 cm and mounted in the SFA in a crossed cylinder geometry. The radius *R* and separation distance *D* of the surfaces at the single point of contact between the cylinders were measured using an optical interferometry technique utilizing the constructive interference of light waves undergoing multiple reflections between the partially reflective Ag coatings^70^. Experiments with surfaces having different *R* were compared by considering the normalized force F(*D*)/*R*, which, according to Derjaguin relation, does not depend on *R*^32^. The distance *D*=0 was defined by the set of wavelengths recorded at mica–mica contact in dry air (before adsorbing the proteins). The error on *F/R* was smaller than 0.1 mN m^−1^ (force detection threshold in the insets of Fig. 7a,b) and the error on *D* about 0.5 nm. Pvfps were adsorbed on both mica surfaces from a 0.02 mg ml^−1^ solution of protein in acid saline buffer containing 10–100 mM acetic acid (with pH≈4 and 3, respectively) and 0.25 M KNO_3_ in purified water (Milli-Q grade from Millipore). Adsorption was stopped after 20 min by rinsing the surfaces with protein-free acidic solution. The homogeneity of the coated mica surfaces was assessed by atomic force microscopy, with r.m.s. roughness of 8.91 and 1.89 Å for Pvfp-3α and Pvfp-5β, respectively (Supplementary Fig. 16). For each pair of mica surfaces we considered at least two contact positions. At each position, we measured at least two force curves *F* versus *D*. Protein adsorption and force measurements were done at a fixed temperature of 25±1 °C.

### Atomic force microscopy

Atomic force microscopy images were acquired in the a.c. mode in air on air-dried samples using an Asylum Cypher S (Asylum, Santa Barbara, USA). A Nanoworld NCSTR-50 cantilever with nominal resonant frequency of 160 kHz and force constant of 7.4 N m^−1^ was used. Samples were scanned at a rate of 1 Hz and 512 points per line resolution in a closed loop set-up.

## Additional information

**How to cite this article:** Petrone, L. *et al*. Mussel adhesion is dictated by time-regulated secretion and molecular conformation of mussel adhesive proteins. *Nat. Commun.* 6:8737 doi: 10.1038/ncomms9737(2015).

## Supplementary Material

Supplementary Information Supplementary Figures 1-16, Supplementary Tables 1-2, Supplementary Note 1 and Supplementary Reference

Supplementary Movie 1 MD trajectory of Pvfp-5β-Dopa homology model. The protein fold is shown in cartoon and all Dopa residues are represented in stick. The disulphide bonds are represented as yellow sticks.

Supplementary Movie 2 Live byssal thread formation and attachment of a Perna viridis green mussel. Snap shots at different time intervals are shown in Supplementary Fig. 14.
