# Supplementary material for: Mussel adhesion is dictated by time-regulated secretion and molecular conformation of mussel adhesive proteins
Source: Nat Commun. 2015 Oct 28;6:8737. doi: 10.1038/ncomms9737 (PMC4640085; doi:10.1038/ncomms9737)
Supplement: Supplementary Information — Supplementary Figures 1-16, Supplementary Tables 1-2, Supplementary Note 1 and Supplementary Reference [file ncomms9737-s1.pdf]

Supplementary Figures

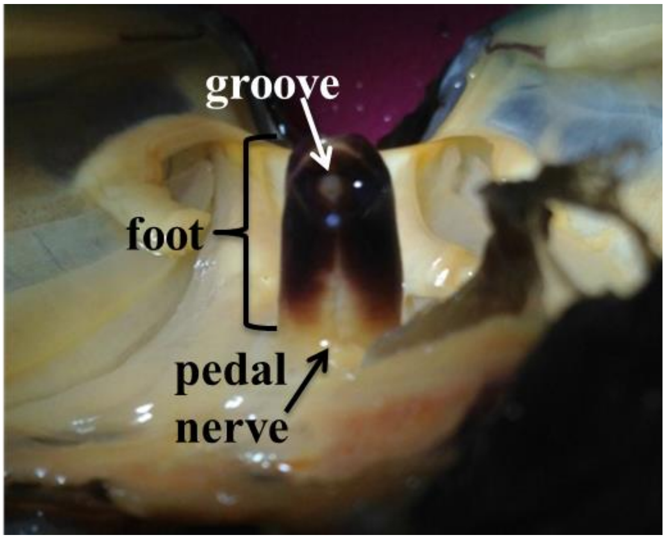

Supplementary Figure 1. KCl solution is injected at the base of the mussel's pedal nerve. Mussel adhesive proteins are retrieved after KCl injection in the mussel's pedal nerve by gently swabbing the groove at the tip of the mussel foot.

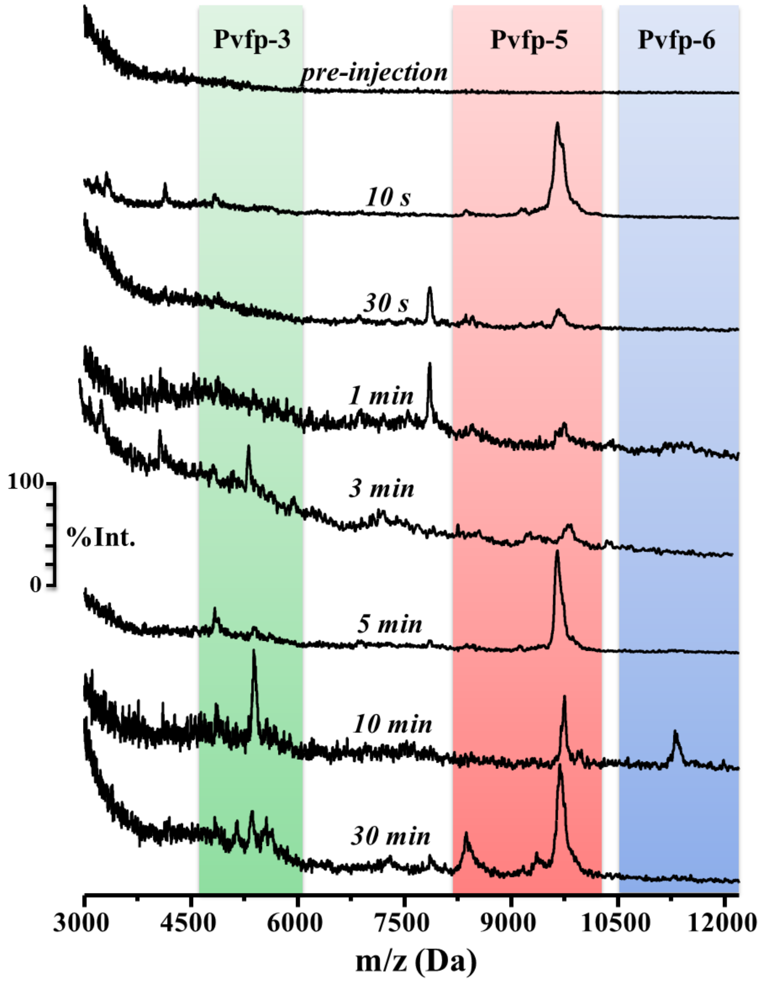

Supplementary Figure 2. MALDI-ToF MS analysis of saline-induced mussel adhesive secretion. MALDI-ToF MS spectra recorded before saline injection and at various time points (from 10 s to 30 min) after injection. Colour areas correspond to Pvfp-3 (green), Pvfp-5 (red), and Pvfp-6 (blue), whereas unlabelled peaks are unidentified compounds.

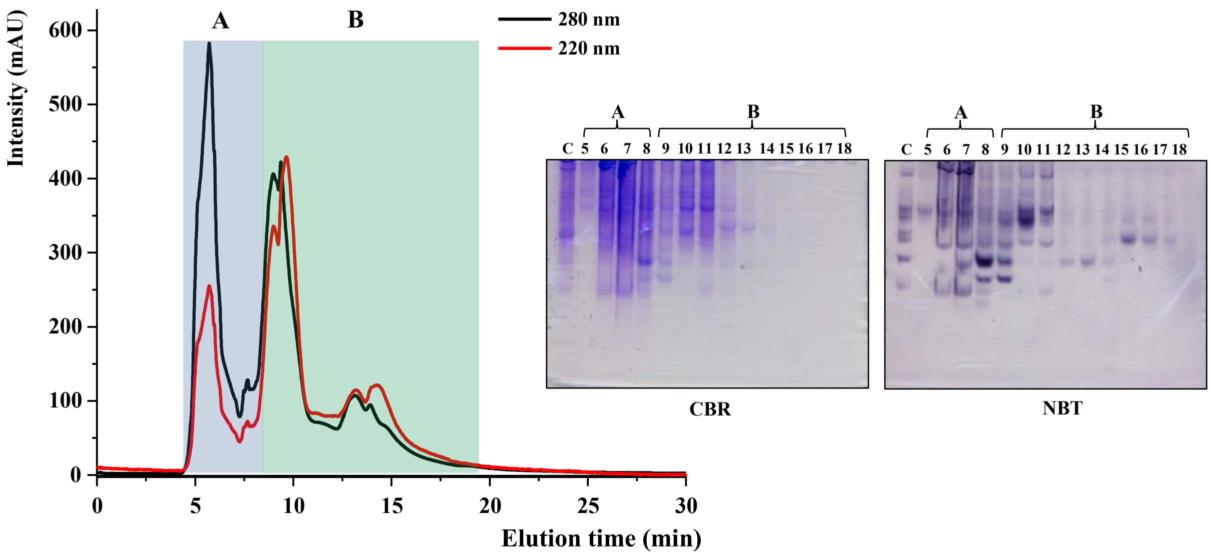

Supplementary Figure 3. Size exclusion (SE) HPLC chromatograms of mussel foot proteins from the KCl-induced secretion. SE-HPLC chromatograms detected at 220 nm (red line) and 280 nm (black line), with fractions pooled at 1 min intervals. Each fraction was loaded onto an AU-gel and stained with CBR and NBT. The two main regions (A and B) in the chromatogram and on the corresponding AU-gels are shown. Numbers on top of the CBR and NBT stained gels indicate fractions pooled from the SE column. The crude extract from the homogenization of the mussel foot is denoted as “C”.

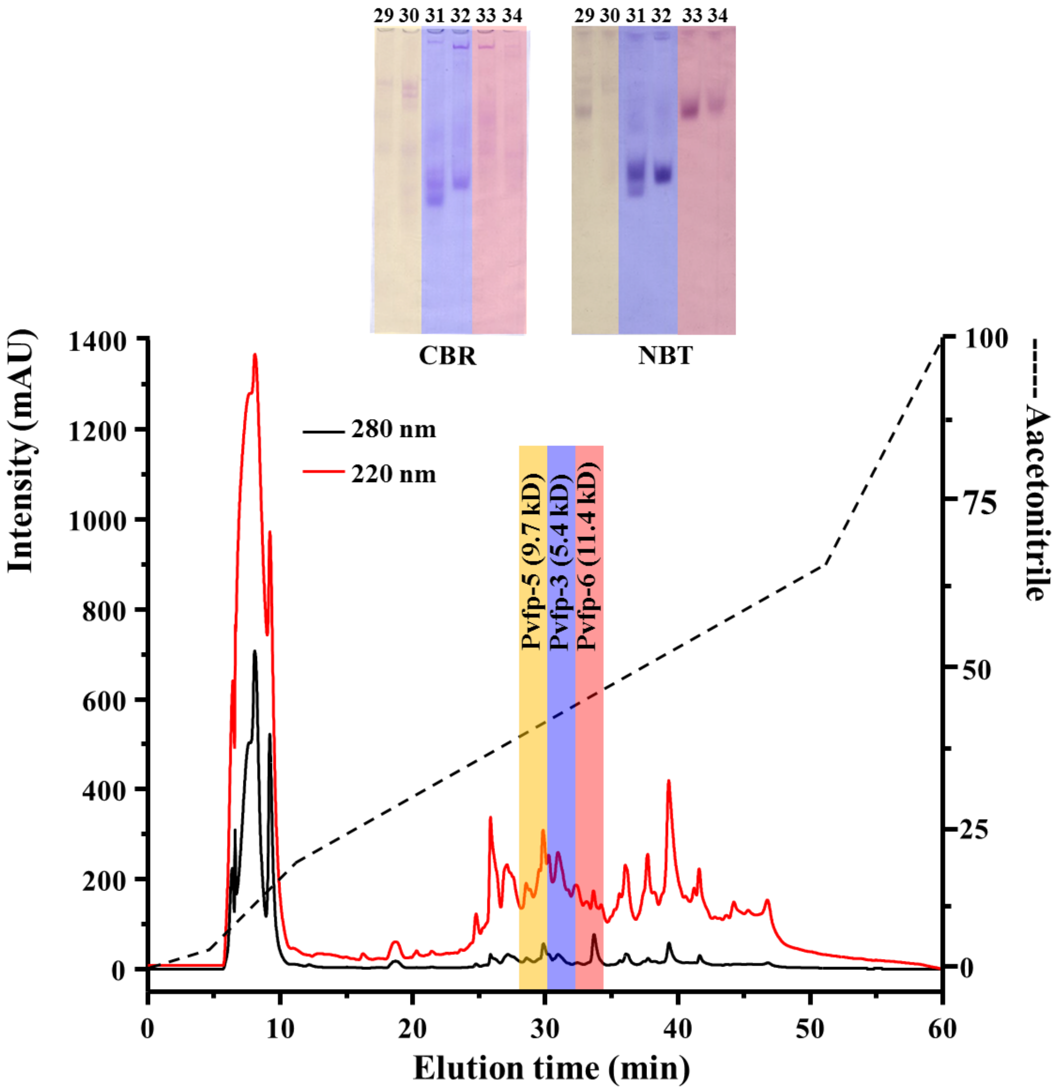

Supplementary Figure 4. Reverse phase (RP) HPLC chromatograms of mussel foot proteins from the KCl-induced secretion. RP-HPCL chromatograms were detected at 220 nm (red line) and 280 nm (black line). A gradient of acetonitrile was employed as shown by the dotted line. All fractions at each minute interval were loaded onto an AU gel and subsequently stained with CBR and NBT to detect proteins and redox-cycling proteins, respectively. Fractions pooled at 29-30 min, 30-31 min, 32-33 min corresponded to Pvfp-5, Pvfp-3, and Pvfp-6, respectively, as indicated in the colour coded regions in the chromatograms and in the corresponding CBR and NBT gels above. Numbers on top of the CBR and NBT stained gels indicates fractions pooled from the reverse phase chromatography column.

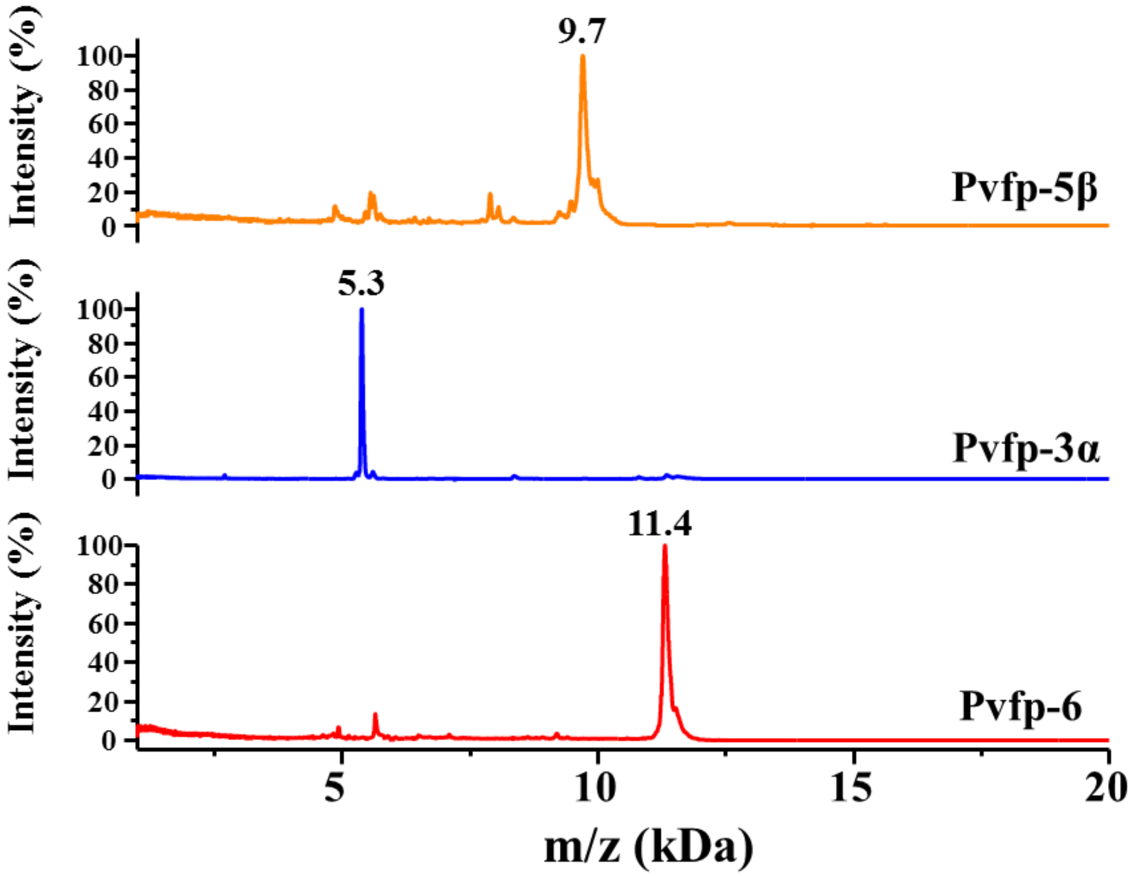

Supplementary Figure 5. MALDI-ToF MS of isolated and purified native Pvfps. MALDI-ToF MS spectra of Pvfp-5β (9.7 kDa), Pvfp-3α (5.3 kDa), and Pvfp-6 (11.4 kDa) purified from size exclusion and reverse phase chromatography.

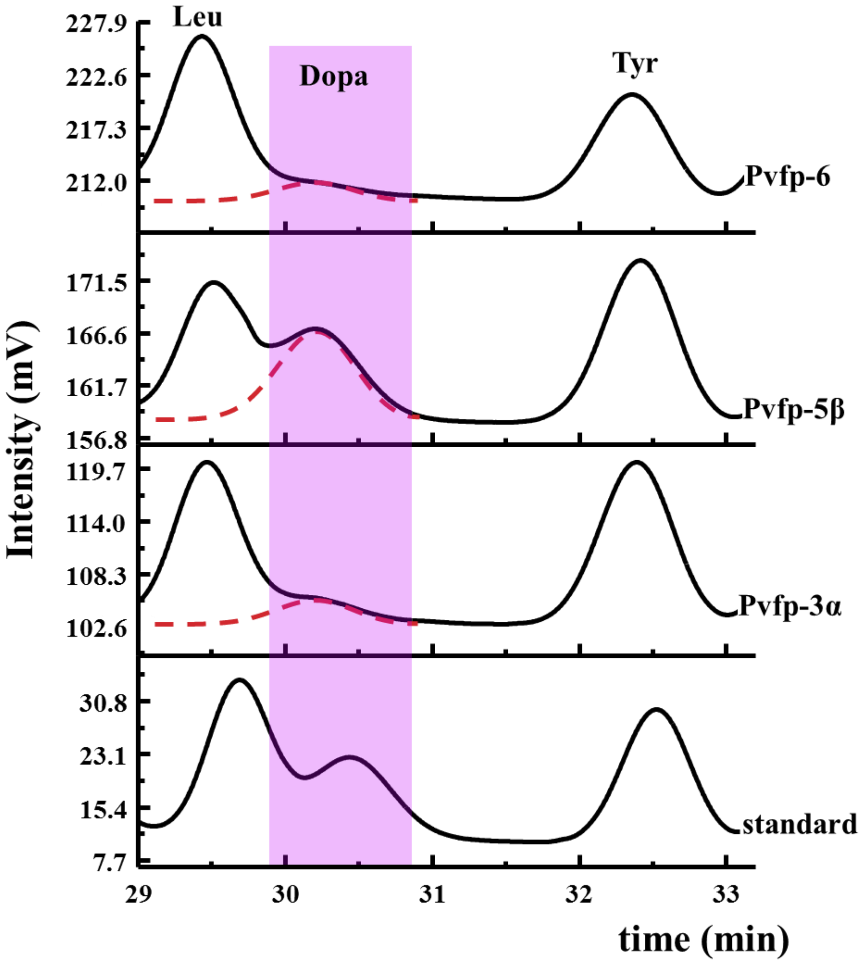

Supplementary Figure 6. Amino acid analysis of Pvfp-5β, Pvfp-3α, and Pvfp-6. The standard mixture of amino acids and Dopa is also shown, along with the elution time for the detection of Dopa (magenta). The red dotted lines outline the area peak component used for Dopa quantification in each Pvfp.

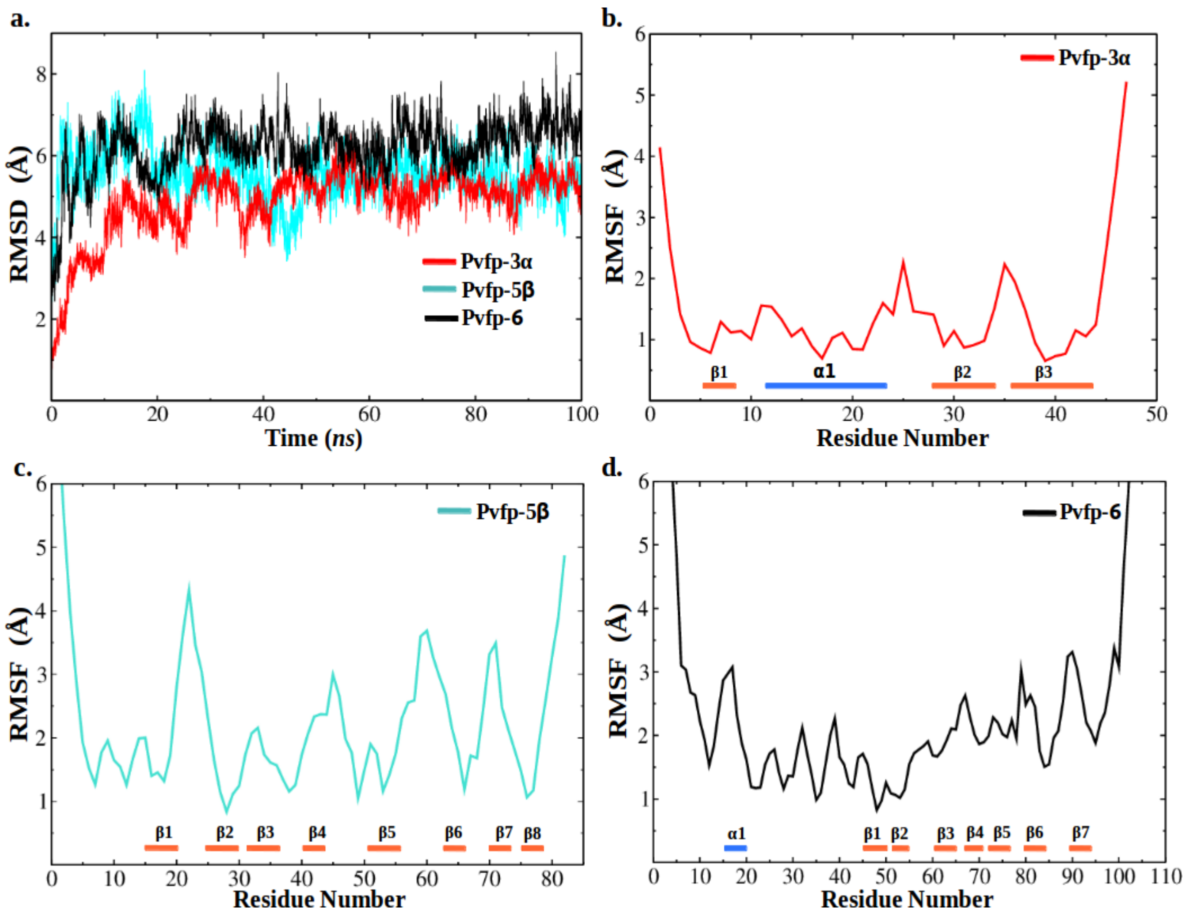

Supplementary Figure 7. Pvfps deviation and fluctuation in MD simulations. (**a**) Root Mean Square Deviation (RMSD) over 100 ns trajectory, and Root Mean Square Fluctuation (RMSF) for (**b**) Pvfp-3α, (**c**) Pvfp-5β, and (**d**) Pvfp-6 with assigned secondary structures.

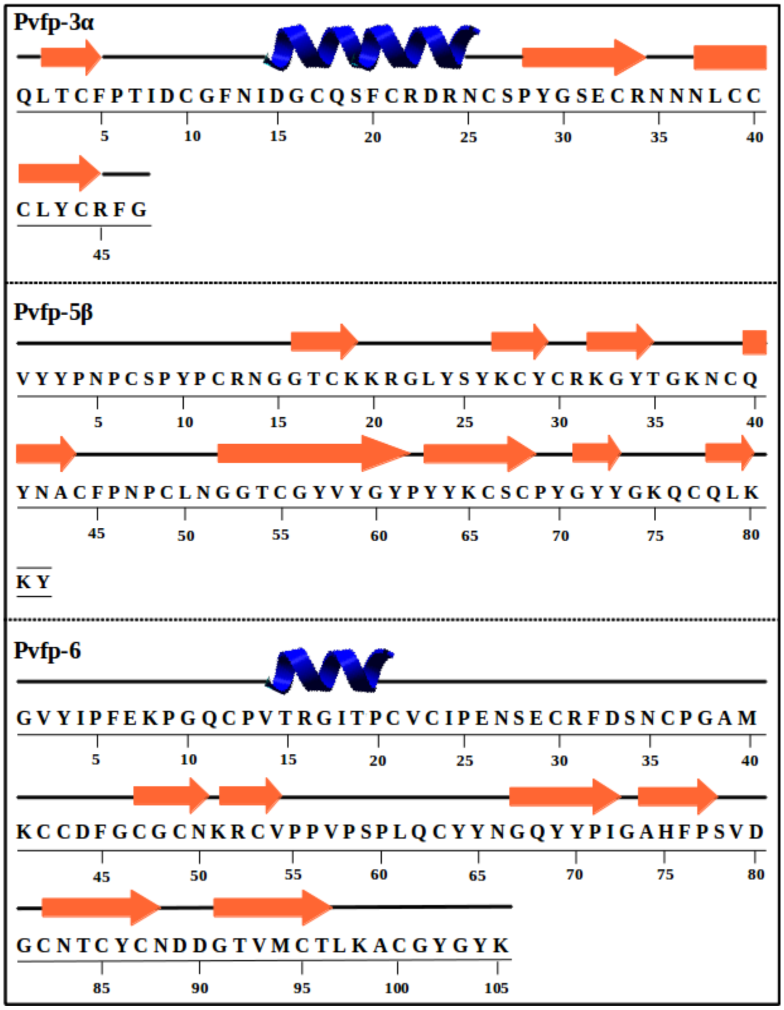

Supplementary Figure 8. Predicted secondary structures of Pvfps. Modelled secondary structures of Pvfp-3α, Pvfp-5β and Pvfp-6 with alpha helixes (blue), *β*-sheets (orange arrows), and random coils (straight lines) on the corresponding amino acid sequences.

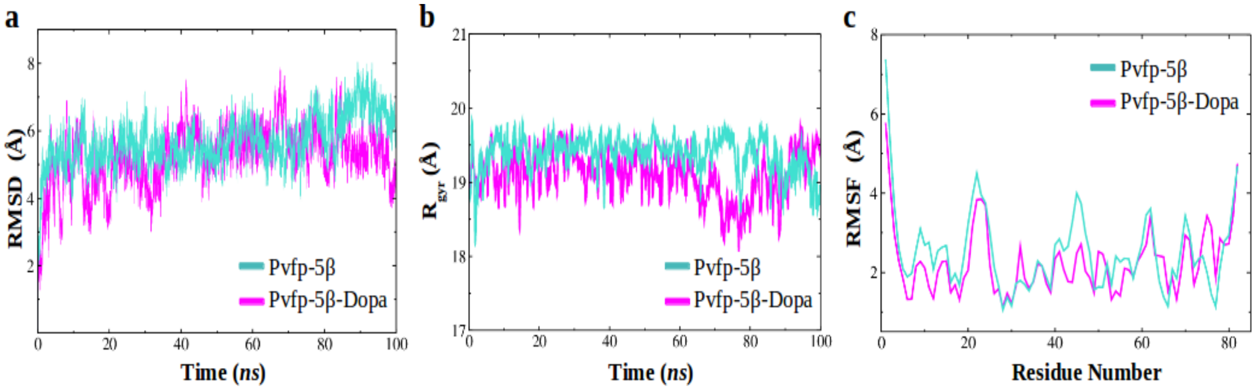

Supplementary Figure 9. Fluctuations of Pvfp-5β and Pvfp-5β-Dopa conformations over MD simulations. (**a**) Root Mean Square Deviation (RMSD), (**b**) Radius of gyration (R_{gyr}), and (**c**) Root Mean Square Fluctuation (RMSF) per residue of conformations sampled during MD simulations Pvfp-5β (cyan) and Pvfp-5β-Dopa (magenta) over 100 ns trajectory.

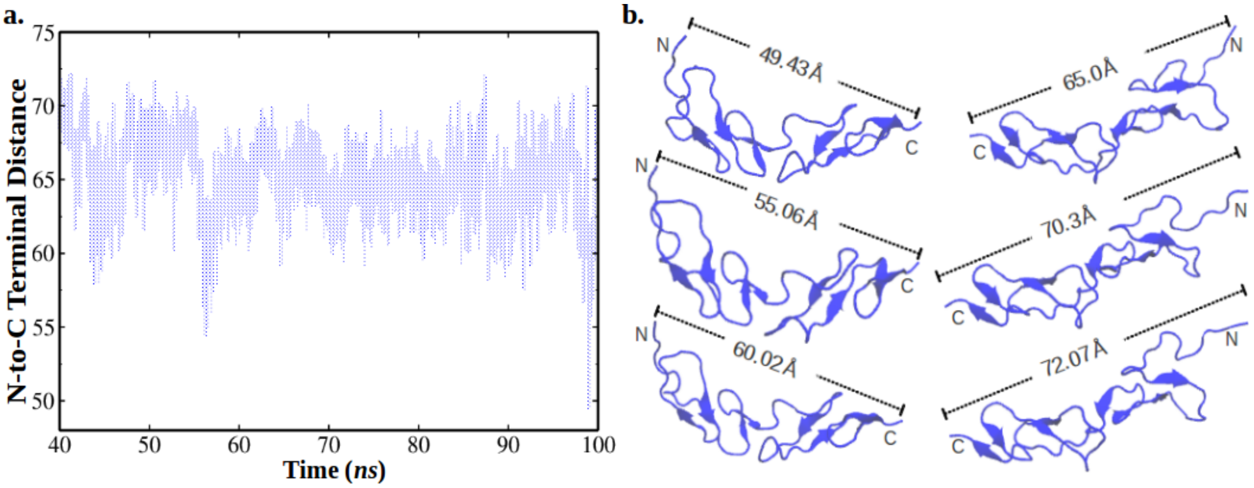

Supplementary Figure 10. The N- to C-terminal distance of Pvfp-5β-Dopa varies along the trajectory of the MD simulations. (**a**) Distance between N- to C-terminal of conformations sampled during MD simulations of Pvfp-5β-Dopa versus simulation time. (**b**) MD snapshots representing a subset of flexible conformations, associated with N- to C-terminal distances.

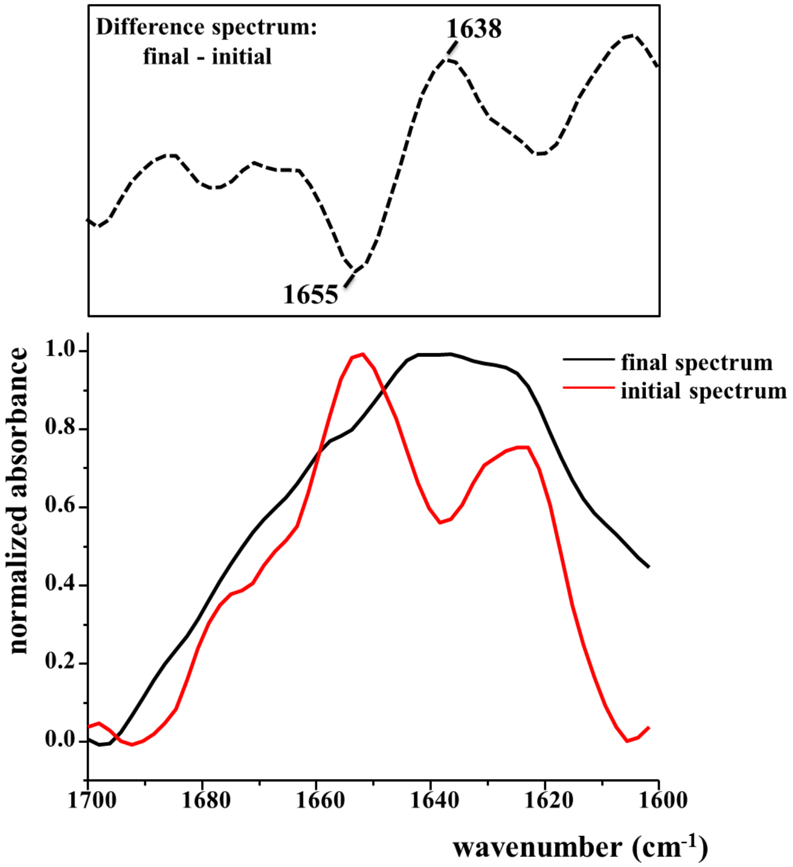

Supplementary Figure 11. Pvfp-5β shows structural rearrangements upon adsorption on TiO_2. Difference spectrum (dashed line) between final (black line) and initial (red line) ATR-IR normalised spectra of Pvfp-5β adsorbed on TiO_2.

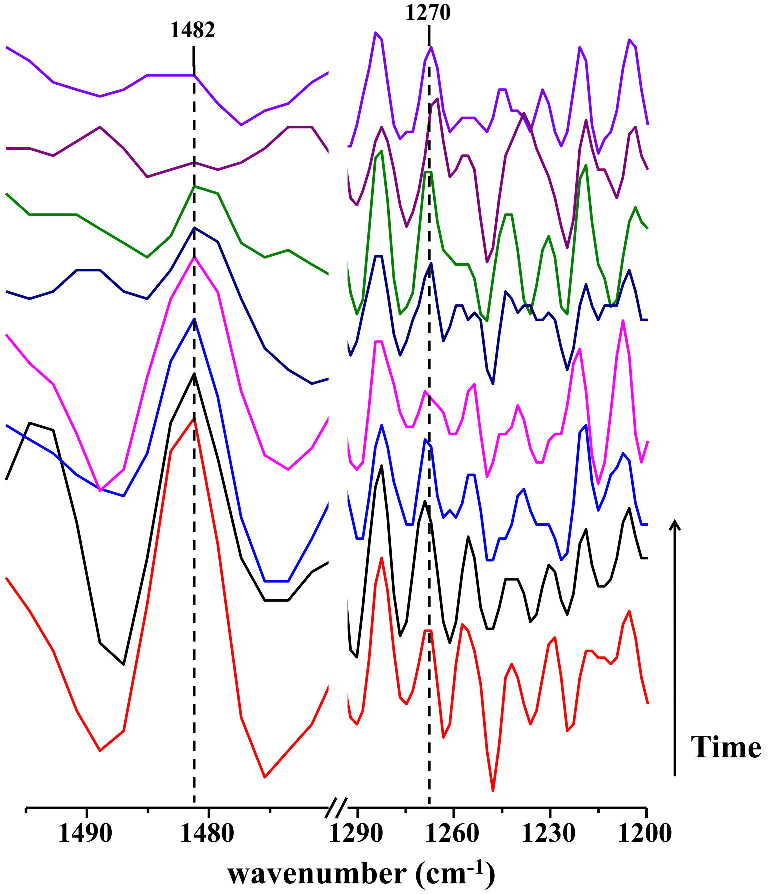

Supplementary Figure 12. Dopa residues in Pvfp-5β coordinate Ti(IV) on TiO_2. Negative second derivative spectra of ATR-IR spectra of Pvfp-5β adsorbed on TiO_2. The position of the characteristic doublet of Dopa/Ti(IV) coordinative bond is shown at 1482 and 1270 cm^{-1}.

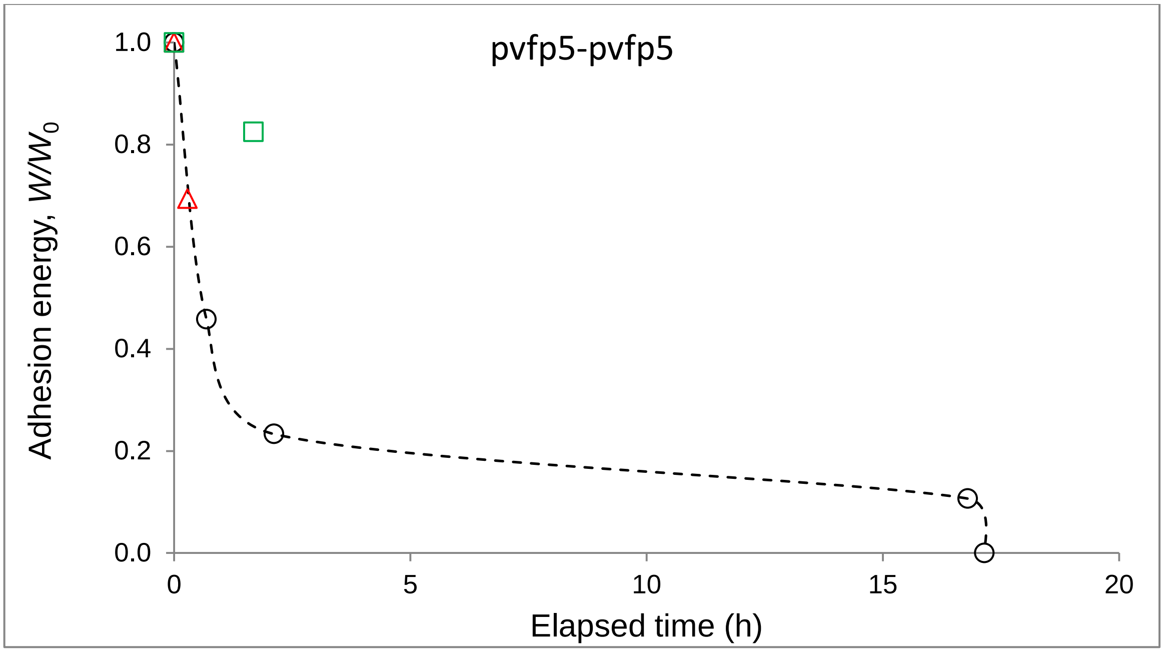

Supplementary Figure 13. Decrease of the normalized adhesion energy W/W_0 of as a function of time, where W_0 is the initial maximum adhesion energy value. The decrease is attributed to Dopa oxidation over time. The dashed line is a guide for the eye.

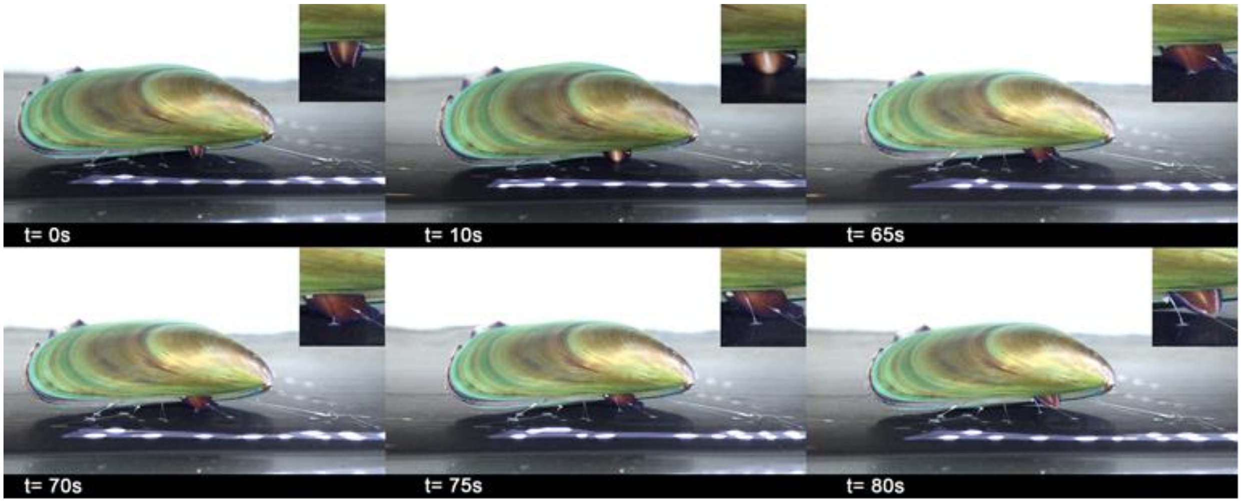

Supplementary Figure 14. A mussel secretes its byssus and adhere to a substrate in about 1 min. Time-lapse of a mussel of *P. viridis* depositing a byssal thread. Insets show higher magnification images of the tip of the mussel foot from which the adhesive plaque and the thread are secreted. The full movie is shown in Supplementary movie 2.

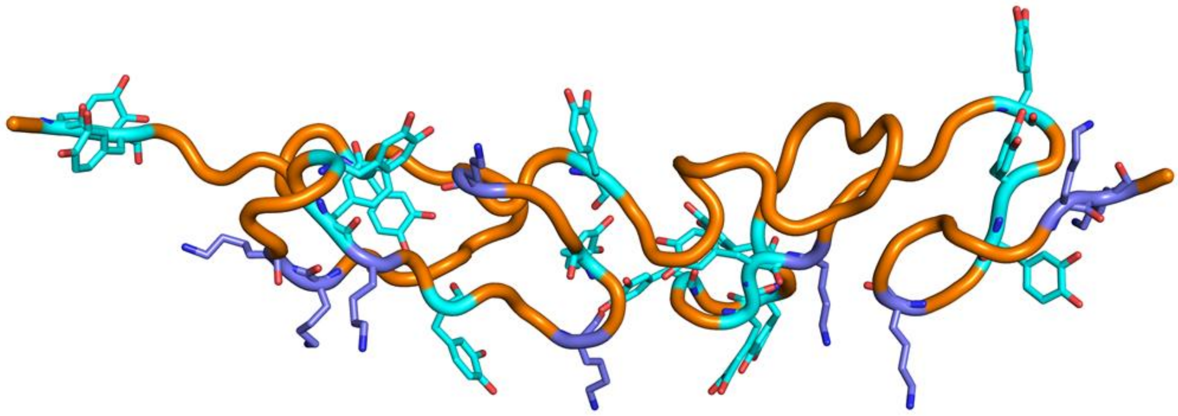

Supplementary Figure 15. Predicted Pvfp-5β secondary structure with positively charged amino acids flanking Dopa residues. Pvfp-5β backbone in orange, Dopa residues in cyan with the two OH groups in red (hydrogen atoms not shown), and lysines in purple.

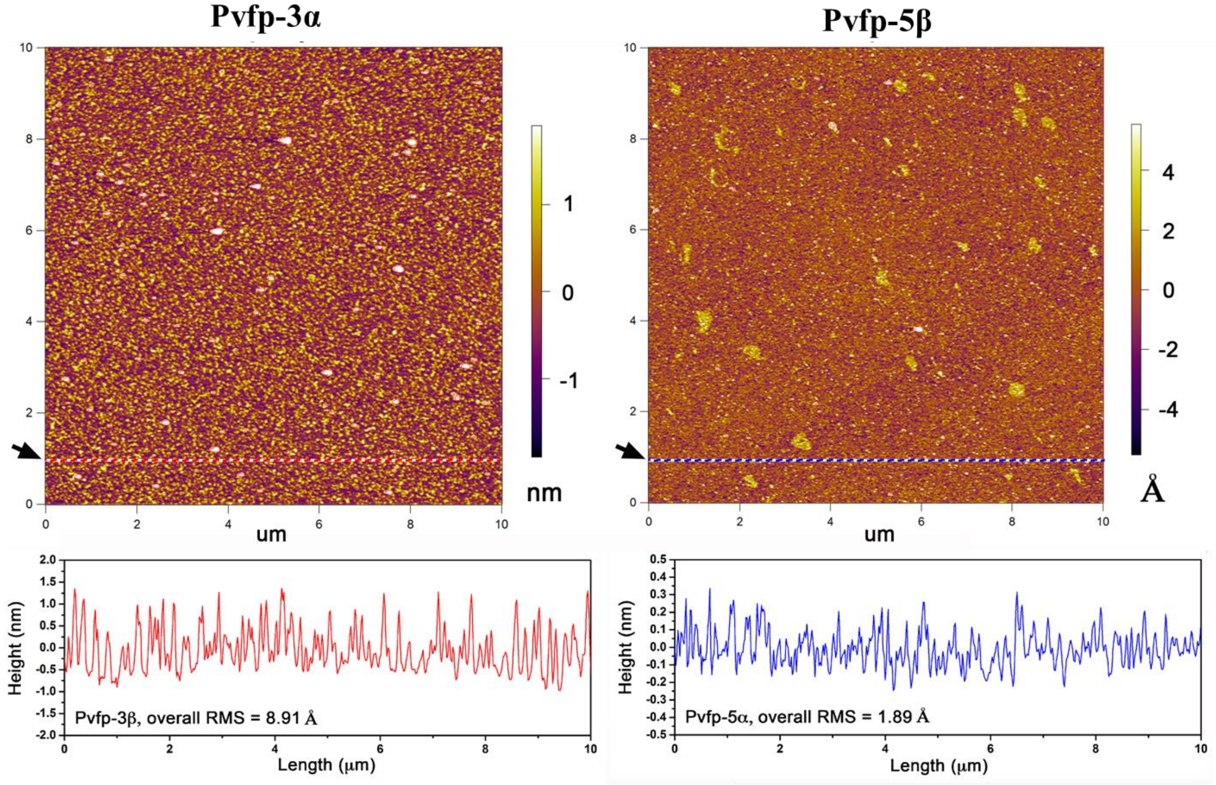

Supplementary Figure 16. Atomic Force Microscopy (AFM) of mussel adhesive proteins on mica. AFM images of dry Pvfp-3α and Pvfp-5β adsorbed from 0.02 mg ml^{-1} solution in 5% acetic acid and 0.25 M KNO_3 on mica. After 20 min adsorption, the mica surfaces were washed with protein-free buffer, and the AFM images show the homogenous distribution of the resulting adsorbed proteins. The height profiles for both proteins are shown in the graphs below, corresponding to the dotted red and blue lines in the respective AFM images (see black arrows).

Supplementary Tables

Supplementary Table 1. Pvfps secreted over the course of the saline-induce mussel secretion. Molecular weight in kDa of Pvfp-3, -5 and -6 variants, as well as other unidentified peaks, detected by MALDI-ToF MS over the time course of the saline-induced adhesive secretion of for *P. viridis* mussels (*n*=15). For comparison, the MALDI-ToF peaks recorded from a mussel adhesive footprint deposited on glass are reported as from Fig. 1a.

Time after saline injection	Pvfp-3	Pvfp-5	Pvfp-6	Others
**10 s**		8.4, 9.1, 9.6, 9.7, 10.1		3.2, 3.3, 4.1, 4.8
**30 s**	5.3, 5.5	8.4, 8.5, 9.6, 9.7		7.8
**1 min**	5.3, 5.4, 5.5, 5.6	9.6, 9.7, 9.8		7.8
**3 min**	5.3, 5.4	9.3, 9.5, 9.7		3.3, 4.1
**5 min**	5.3, 5.4, 5.5, 5.6	9.1, 9.3, 9.5, 9.6	11.3	3.2, 3.3, 4.7, 4.8, 7.8
**10 min**	5.4, 5.5, 5.7	9.1, 9.3, 9.7	11.3	4.7
**30 min**	4.8, 5.1, 5.3, 5.4, 5.5, 5.7	8.4, 8.5, 9.3, 9.7	11.3	7.8
**Native adhesive footprint**	5.2, 5.4, 5.6,	8.4, 9.1, 9.3	11.3	

Supplementary Table 2. Protein mass secreted during the saline-induced secretion. Average protein mass with standard deviation (sd) for the time-resolved induced adhesive secretion of Pvfps from *n*=15 mussels. The Pvfps detected by MALDI-ToF MS are also reported at each time interval. Pvfps that are only occasionally detected at the corresponding time intervals are indicated in parenthesis**.**

time interval	mass (µg) ± sd	Pvfps
0-10 s	5.79 ±0.48	5
10 s-30 s	13.69 ±1.97	5 (3)
30 s-1 min	21.65 ±2.39	3, 5
1 min-3 min	30.80 ±3.72	3, 5
1 min-5 min	40.18 ±4.84	3, 5
5 min-10 min	49.19 ±6.37	3, 5, 6
10 min-30 min	56.03 ±6.45	3, 5, 6

Supplementary Notes

Supplementary Note 1: Aggregates in Pvfps

DLS measurements (Fig. 2c) showed that Pvfps at 0.1 mg ml^{-1} and 0.25 M KNO_3 formed aggregates, with hydrodynamic diameters larger than the size of single proteins. The diameter d of a compact (*i.e.* non polymeric or non-linear) aggregate typically increases with the number n of building blocks in the aggregate as $d \sim n^{1/3}d_0$, where d_0 is the size of a block (the volume is $v_0 = d_0^3$)[1]. The number of proteins in an aggregate will be $n \sim d_H^3/d_{SIM}^3$, where d_H^3 is approximately the volume of an aggregate as measured by DLS and d_{SIM}^3 is the volume of a single protein obtained from simulations. Hence, about 21, 9, and 126 proteins comprise the aggregates measured for Pvfp-3α, Pvfp-5β and Pvfp-6, respectively. In other words, Pvfp-6 aggregates contain about 6- and 14-fold more molecules than Pvfp-3α and Pvfp-5β aggregates, respectively, indicating a different mechanism of protein-protein interaction. The surface electrostatic potential is notably distinct among Pvfps (Fig. 4), with Pvfp-3α Pvfp-6 exhibiting positively and negatively charged domains, whereas Pvfp-5β presents only a positively charged region. These disparities in Pvfps' charge surface distribution likely account for the different aggregation behaviour in solution and for the variation in adsorption behaviour on surfaces. For instance, Pvfp-6 aggregates in solution may originate from stronger charge-charge interactions, leading to larger aggregates that those for Pvfp-5β, which is mostly positively charged.

Supplementary Reference

1. Rubinstein, M. & Colby, R. *Polymer Physics*. Oxford University Press, Oxford, UK (2003).
